# Complete larval development of the Monkey River Prawn *Macrobrachium lar* (Palaemonidae) using a novel greenwater technique

**DOI:** 10.1186/2193-1801-3-568

**Published:** 2014-09-30

**Authors:** Monal M Lal, Johnson Seeto, Timothy D Pickering

**Affiliations:** Centre for Sustainable Tropical Fisheries and Aquaculture, James Cook University, Townsville Campus, Townsville, Queensland Australia; College of Marine and Environmental Sciences, James Cook University, Townsville Campus, Townsville, Queensland Australia; School of Marine Studies, Faculty of Science, Technology and Environment, University of the South Pacific, Laucala Campus, Suva, Fiji Islands; Coastal Fisheries Programme, Aquaculture Section, Secretariat of the Pacific Community, Suva Regional Office, Nabua, Suva, Fiji Islands

**Keywords:** Biofloc, Greenwater technique, *Macrobrachium lar*, Zoea, Larvae, Decapodid, Larval development

## Abstract

This study documents the complete larval development of the Monkey River Prawn *Macrobrachium lar* using a new greenwater rearing technique. Approximately 6,000 larvae were reared for 110 days at an initial stocking density of 1 ind./6 L. Salinity at hatch was 10 ± 2 ppt and progressively increased to 30 ± 2 ppt until decapodids had metamorphosed. Temperature was maintained at 28 ± 0.5°C, pH at 7.8 ± 0.2, DO_2_ > 6.5 mg/L and NH^4+^ and NH_3_ ≤ 1.5 and ≤0.1 ppm respectively throughout the culture period. Larval development was extended and occurred through 13 zoeal stages, with the first decapodid measuring 6.2 ± 0.63 mm in total length observed after 77 days. 5 decapodids in total were produced, and overall survival to this stage was 0.08%. Overall, the pattern of larval growth shares similarities with those of other *Macrobrachium* spp. that have a prolonged/normal type of development, and it is likely that larvae underwent mark time moulting which contributed to the lengthened development duration. While this study represents a significant breakthrough in efforts to domesticate *M. lar*, improvement of larval survival rates and decreased time till metamorphosis are required before it can become fully viable for commercial scale aquaculture.

**Electronic supplementary material:**

The online version of this article (doi:10.1186/2193-1801-3-568) contains supplementary material, which is available to authorized users.

## Introduction

The Monkey River Prawn *Macrobrachium lar* is a large palaemonid freshwater prawn indigenous to a number of regions across the Indo-West Pacific, and of importance to artisanal and subsistence fisheries in almost all areas where it occurs (Holthuis 1950, 1980; Nandlal 2005, 2010; Barbier *et al.*^2006^; Ponia 2010).

Because of its size, relatively fast growth rates and a number of other favourable culture characteristics, it appears to have good potential for aquaculture except for one major constraint; the availability of seed stock for grow-out is severely limited by difficulties in rearing the larvae from hatch until metamorphosis into the decapodid. Shokita *et al.* (1984) in their report on inland water prawns present in Fiji identified 3 species (*M. lar*, *M. australe* and *M. equidens*) which appeared to have potential for aquaculture in the country, and went on to state that mass artificial seed production of any had yet to succeed. Of the three species discussed, *M. lar* was put forth as the leading candidate owing to the large size it attains relative to the other two species.

The state of knowledge on the larval development of *M. lar* is fragmentary, largely due to difficulties encountered by previous researchers in rearing larvae to metamorphosis. The first attempt at completing larval development in captivity was by Kubota (1972), who was able to rear larvae till the fifth zoeal stage. Further attempts were made by Atkinson (1973, 1977), Muranaka in Hanson and Goodwin (1977), Takano (1987), Nandlal (2010), Sethi and Roy in Kutty and Valenti (2010) and Sethi *et al.* (2011), however all experienced total larval mortality before metamorphosis to the decapodid. There is also a distinct lack of further information resulting from the work of M. S. Muranaka (in Hanson and Goodwin 1977), who had reportedly managed to produce decapodids, however it appears no further publications resulted from that study.

These studies provide some indications why larval rearing so far has proved to be unsuccessful, and include conditions of improper salinity, temperature, disease (Kubota 1972), as well as nutrition (Kubota 1972; Atkinson 1973, 1977). The most recent investigation into the role of salinity and temperature on larval development found that newly-emerged larvae are able to tolerate fresh or brackish water of approximately 10 ppt, but require gradually increasing salinities post-hatch reaching 30–35 ppt, which needs to be maintained until metamorphosis into the decapodid (Lal *et al.*^2012^). The information obtained from these investigations was utilised in an attempt to close the lifecycle for this species, and to concurrently better understand its early life history.

A summary of developmental characteristics and rearing condition requirements of *Macrobrachium* spp. whose larviculture has been investigated is provided in Shokita (1985) and Willführ-Nast *et al.* (1993). Among species which exhibit the typical or prolonged/normal type of development, there are comparatively few which like *M. lar*, require oceanic salinities for successful development. This paper describes the morphological larval development of *M. lar* which was successfully completed in the laboratory using a novel greenwater rearing technique, with the specific objective of describing larvae in a simple manner through all larval developmental stages until metamorphosis into the decapodid, in order to facilitate further rearing efforts for aquaculture.

## Methods

### Broodstock collection and maintenance

8 adult male and 19 female *M. lar* broodstock were collected at 2 sites in Waisere Creek, Vugalei District, Tailevu Province, Viti Levu, Fiji (17° 56’ 42.14” S; 178° 33’ 11.31” E and 17° 56’ 43.42” S; 178° 32’ 55.81” E). All prawns were maintained in single 2500 L rectangular Fibre-Reinforced Plastic (FRP) and square 1000 L polyethylene tanks, filled to a depth of 500 mm with 50 μm filtered freshwater maintained at 26 ± 0.5°C. Aeration was provided via four air diffusers at ~200 mL/sec at each outlet. Water parameters remained at DO_2_ > 6.5 mg/L and pH 7.2-7.6. Females bearing mature grey coloured eggs were transferred to hatching tanks.

### Hatch tank preparation

Circular 1000 L flat-bottomed polyethylene tanks for hatching larvae doubled as larval rearing tanks (LRTs). Approximately 300 L brackish water (10 ± 0.5 ppt at 28 ± 0.5°C) which had been filtered to 50 μm was prepared in a dedicated 1000 L polyethylene water preparation tank. Other water parameters were as follows; pH 7.8 ± 0.2, DO_2_ > 6.5 mg/L and average NH^4+^ and NH_3_ ≤ 1.5 and ≤0.1 ppm respectively. Gentle aeration was provided at ~15–30 mL/sec via four air diffusers. A fluorescent light tube fitting with a 1.2 m Osram 36 W ‘warm white’ tube and 1.2 m Eurolux 36 W ‘cool white’ tube was suspended over each tank providing ~6700 lx at the surface of the water. These lights were kept off during hatching and turned on the morning after.

### Rearing of larvae

Larvae were reared in a mass culture trial over 110 days. 5 ovigerous females at the grey egg stage were introduced into 3 separate LRTs and maintained for 48 h until they had spawned. Females were not fed during this period. An initial hatch estimate was made after 24 h, and the tank volume increased by 100 L using brackish water mixed to 20 ± 0.5 ppt. This strategy was employed to slowly increase the salinity of the culture medium, and tank volume was increased by 100 L without any water exchanges every 24 h until 800 L was reached. This approach was developed after Lal *et al.* (2012). After 800 L was reached, 12-25% (~100-200 L) was exchanged daily with 50 μm filtered seawater at 30 ± 0.5 ppt and 28 ± 0.5°C, depending on requirements. The target salinity when mixing replacement water was set at either 20 or 32 ppt. During the first few days of culture, replacement water was mixed to 20 ppt, to progressively increase salinity with successive daily water exchanges. Because larvae were hatched at 10 ppt, ~20 ppt was attained by day 7 of culture.

Following this, all subsequent water exchanges for each tank were mixed to 32 ppt, attaining ~30 ppt by day 30 of culture. This salinity was maintained until decapodids were produced, after which point all water exchanges used treated freshwater to progressively reduce salinity to 0 ppt. Because larvae were reared using a greenwater technique, at 2–3 day intervals the replacement water for water exchanges was sourced from cultures of either marine or freshwater microalgae or both. Both sets of cultures were of mixed species of varying concentrations, as it was found to be much easier and less time-consuming to mass culture using this method than to maintain monospecific/axenic cultures of different species due to the volumes required.

In the marine microalgae cultures referred to as ‘brown water’ (Imamura *et al.*^2009^), various species of diatoms were found to dominate, with the genera *Nitzschia*, *Navicula* and *Skeletonema* being the most common. Cells of these diatoms were naturally present in the seawater used for the cultures, and were seedstock for the starter cultures. Likewise with the freshwater microalgae cultures which were also of mixed species and referred to as ‘green water’ , a single *Desmodesmus* sp. and several *Chlorella* spp. were dominant. The source of the stock green water was a series of three 5000 L tanks containing Nile Tilapia *Oreochromis niloticus*. By using a mixture of brown water and green water mixed to the required target salinity, the formation of biofloc particles was encouraged in the tanks which larvae were seen to actively feed on. Further descriptions of methods used to culture the brown and greenwater are documented by Imamura *et al.* (2009).

Aeration volume was progressively increased as the larvae developed. Gentle aeration was employed at ~15–30 mL/sec at each air diffuser for early stage larvae *e.g.* zoea I, so as not to damage them by excessive turbulence. This rate was increased to ~150–200 mL/sec for mid and late stage larvae (zoeae V to X), in order to keep feed and biofloc particles in suspension where they could be accessed. This also prevented circulating matter from settling on the tank floor which would have lead to accelerated decomposition and poor water quality.

### Feeding

**Figure 1:**
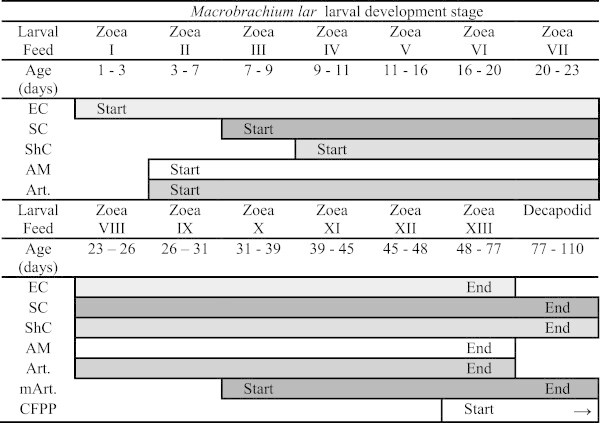
**Larval feeding schedule.** The feeds offered were Egg custard (EC), squid custard (SC), shrimp custard (ShC), Algamac 3050 flake (AM), *Artemia* nauplii (Art.), *Artemia* meta-nauplii (mArt.) and commercial formulated prawn pellet (CFPP).

Larvae were fed *ad lib.* at 2-hourly intervals, from 0700 to 1900 daily with 3 different types of steamed custard feeds (Table 1). These were offered by pressing the custard particles through sieves of varying mesh sizes as outlined in Figure 1, with *Artemia* nauplii being offered once daily in the afternoon. The sieve mesh sizes were selected according to the development stage of larvae in individual tanks, to ensure they were able to capture and feed on particles of an appropriate size in relation to their body and mouth sizes. The feeding sieve mesh sizes are detailed in Table 2.

**Table 1:** **Prepared custard feed ingredients modified from Imamura** ***et al.*** **(** 2009 **)**

Egg custard	Squid custard	Prawn custard
1 egg yolk (approx. 20 g in weight)	25 g whole squid^b^ flesh (including viscera)	25 g whole prawns^c^
20 g milk powder	25 g whole prawns	1 EPA capsule (1000 mg)^a^
15 mL water	1 whole egg	1 multivitamin capsule
1 EPA capsule (1000 mg)^a^	1 egg yolk (approx. 20 g in weight)	10 mL soyabean cooking oil
1 multivitamin capsule	1 EPA capsule (1000 mg)^a^	15 g Algamac 3050 flake
10 mL soybean cooking oil	1 Multi-vitamin capsule	
	10 mL soybean cooking oil	
	1 lecithin capsule (1200 mg)	

^a^EPA 1000 mg capsules included 180 mg EPA and 120 mg DHA.

^b^Whole Boston Squid *Loligo pealei* (Lund's Seafood Inc.).

^c^ *Palaemon concinnus, P. debilis, Macrobrachium grandimanus* and *M. equidens*.

**Table 2:** **Feeding sieve mesh sizes used for particular larval stages**

Feeding sieve number	Feeding sieve mesh size (μm ^2^)	Larval stages
1	150	zoea I to zoea III
2	400	zoea II to zoea V
3	750	zoea VI to zoea X
4	1000	zoea X to decapodid

When larvae had developed to zoea XIII, crushed formulated prawn pellet (32% crude protein, Crest Chicken Limited, Fiji) was offered to complement the custard feeds. Algamac 3050 flake (Aquafauna Biomarine Inc, Hawthorne, USA) was also used to supplement the custard feeds and to enrich *Artemia* nauplii offered to the larvae. For direct feeding, the Algamac 3050 flake was weighed according to the daily feed ration measured for each tank and either screened through the appropriately sized feeding mesh for zoeae I to IX larvae, or added directly to the water for zoea X onwards.

Larvae were observed consuming a number of other live feed items apart from *Artemia* nauplii and meta-nauplii. The majority of these comprised of biofloc, and biofloc-associated microorganisms. A number of the biofloc-associated microorganisms included various types of rotifers, the most abundant of which was a *Colurella* sp. (Family Brachionidae), together with various nematodes and protozoans.

It proved to be difficult to quantify biofloc volumes, however a general guide established was to maintain concentrations of 1500–2500 pieces of biofloc/L. This proved to be an apparently optimal density based on qualitative observations of larval feeding behaviour. A single piece of biofloc was loosely defined as any aggregation of biofloc material up to 5 mm. At times between scheduled feeding intervals if larvae were observed to have consumed all feed from the previous offering, they were encouraged to feed on biofloc present in the tank by stirring settled material on the tank floor.

### Larval microscopy

All microscopy was carried out using a binocular compound microscope (Olympus CH-2) fitted with a calibrated eyepiece graticule. All photomicroscopy was carried out using a digital camera (Nikon Coolpix E995) mounted on one of the microscope eyepieces. Larvae were routinely examined at 0900 daily, using a cavity slide without a coverslip to avoid squashing the specimens. All observations and photographs were of live individuals and specimens were either preserved immediately afterwards in 80% ethanol for larval staging work, or returned to the tank.

### Description of larval development in *Macrobrachium lar*

A select number of larval morphological features were examined and changes in these recorded and used for characterising larval development. These morphological features included carapace armature (rostrum, supra-orbital spines and pterygostomian spines), as well as developments of the tail fan (telson and uropods), pereiopods (walking legs), pleopods (swimmerets) and antennules and antennae. Larvae were also measured to determine their total and carapace lengths.

10 individuals of the same apparent morphological developmental stage and age were sampled and used in making determinations of larval stage and average size. All larvae were thoroughly examined to ensure the morphological features being recorded were consistent between the individuals sampled. Once determinations of stage had been made, representative specimens were lodged at the Marine Reference Collection of the School of Marine Studies, Faculty of Science, Technology and Environment, University of the South Pacific, Suva, Fiji Islands under Catalogue Number 5940.

Descriptions of the morphological development of *M. lar* larvae provided here have intentionally been kept simple, for the purpose of easily identifying developmental stages. Rather than adopting the approach of providing an exhaustive description of larval morphological features, descriptions are from a more practical perspective and have used a few, major and easily detectable features rather than minor morphological changes. The rationale behind this was to provide a means of simply and rapidly identifying live specimens for any future larviculture work aimed at mass production of decapodids in a hatchery system, involving this or a similar species.

### Specimen drawings

Simple line diagrams of specimens showing the body outlines without internal structures e.g. organs, musculature and external chromatophore patterns were produced with the aid of photographs of live specimens. All diagrams were then outlined in black ink before being scanned at 600 dpi and processed using Adobe Photoshop version 7.0 software.

## Results

### Larval rearing

**Figure 2:**
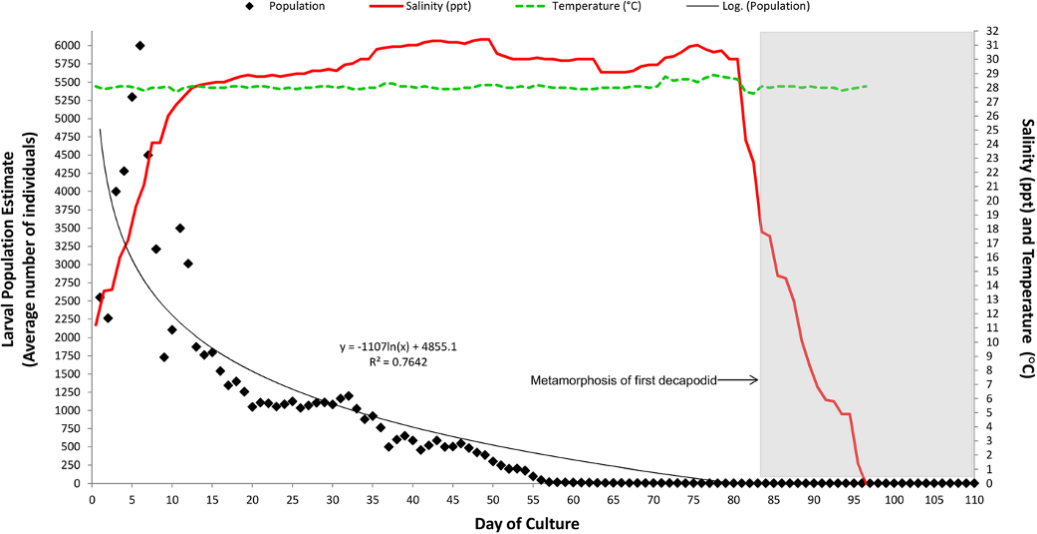
**Larval survivorship, salinity and temperature data recorded over the culture period.** The increase in larval population over days 1 to 6 is due to continued input of larvae from spawning broodstock. Broodstock were removed on day 6. Following metamorphosis of the first decapodid, the water parameters displayed here were recorded in the tank containing the remaining larvae.

Larvae developed through 13 zoeal stages before metamorphosing (Table 3), with 5 decapodids produced after 77, 78, 85, 101 and 110 days of culture respectively. Survival to this stage was 0.08%, and 0.27% to zoea XII/XIII. Mortality proved to be very high, especially during the first few days of culture (Figure 2).

**Figure 3:**
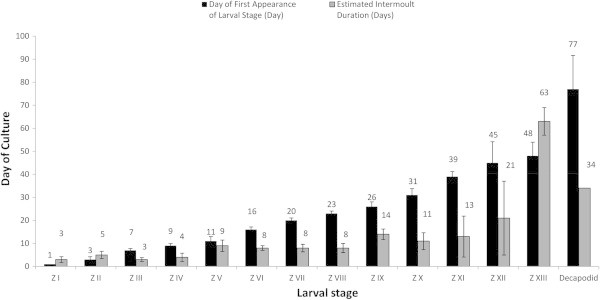
**Day of first appearance of larval stages and their intermoult durations.** Values indicated are ± Standard Deviation (SD).

Patterns of growth were regular until zoea V was reached (Figure 3). Development through zoeae I to IV occurred consistently with average intermoult durations of ~3 days, ~8 days from V to VIII and ~12 days from IX to XI. From this point, development was irregular with durations of 21 and 63 days for zoeae XII and XIII respectively. Metamorphosis into the decapodid was also prolonged, taking 34 days from the time of metamorphosis of the first till last individuals. Salinity and temperature variations over the culture period did not vary outside the desired limits (Figure 2).

**Table 3:** **Age and size ranges of** ***M. lar*** **larvae**

Stage	Age (Day of first appearance)	Carapace length (mm)	Total length (mm)
Zoea I	1	0.25 ± 0.20	0.8 ± 0.21
Zoea II	3	0.35 ± 0.10	1.1 ± 0.32
Zoea III	7	0.40 ± 0.10	1.2 ± 0.25
Zoea IV	9	0.45 ± 0.15	1.3 ± 0.38
Zoea V	11	0.50 ± 0.20	1.5 ± 0.28
Zoea VI	16	0.70 ± 0.15	2.7 ± 0.36
Zoea VII	20	0.65 ± 0.25	2.9 ± 0.42
Zoea VIII	23	0.80 ± 0.22	3.2 ± 0.31
Zoea IX	26	1.18 ± 0.12	4.15 ± 0.29
Zoea X	31	1.20 ± 0.15	4.2 ± 0.32
Zoea XI	39	1.20 ± 0.18	4.4 ± 0.28
Zoea XII	45	1.65 ± 0.26	5.0 ± 0.37
Zoea XIII	48	1.80 ± 0.32	5.4 ± 0.41
Decapodid	77	2.25 ± 0.38	6.2 ± 0.63

### Metamorphosis

Due to poor larval survival (10 individuals remaining by day 65), it became impractical to rear survivors in the single 1000 L tank which had not encountered total mortality. All larvae were then transferred to a 60 L cylindro-conical fibreglass LRT and the trial continued. After the first decapodid was observed on day 77, salinity was reduced from 30 to 24.3 ppt over days 80–81, with further gradual reductions carried out until 0 ppt was reached by day 96.

The first decapodid was removed and transferred to a separate 60 L cylindro-conical fibreglass LRT, which was maintained at 28.8 ppt and 28 ± 0.5°C for a period of 24 h, before salinity was reduced to 0 ppt in 5 ppt steps each day by exchanging 20–25% of the tank volume (Figure 2). This procedure was also carried out for the next 4 decapodids collected from the tank.

### Descriptions of larval development stages

**Figure 4:**
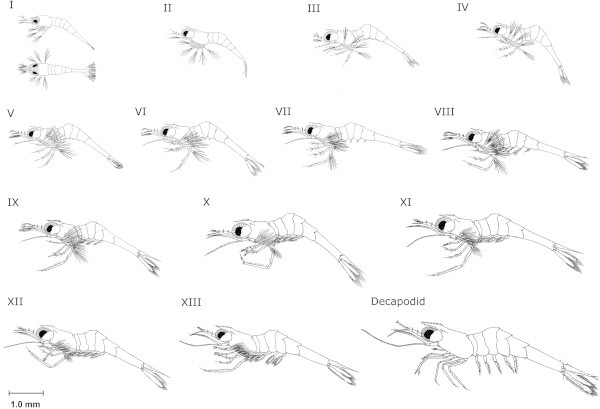
**Larval development stages of** ***M. lar*** **.**

A summary of larval stages and their developmental features is presented in Table 4, and the appearance of each larval stage is reproduced in line diagrams in Figure 4. Photomicrographs showing specific larval staging features for each zoeal and the decapodid stage are presented in Additional file 1: Figure S1, Additional file 2: Figure S2, Additional file 3: Figure S3, Additional file 4: Figure S4, Additional file 5: Figure S5, Additional file 6: Figure S6, Additional file 7: Figure S7, Additional file 8: Figure S8, Additional file 9: Figure S9, Additional file 10: Figure S10, Additional file 11: Figure S11, Additional file 12: Figure S12, Additional file 13: Figure S13, Additional file 14: Figure S14, Additional file 15: Figure S15.

**Table 4:** **Summary of readily discernible features characterising the larvae and decapodid of** ***M. lar***

Stage	Eyes	Rostrum (Teeth)	Antennal flagellum (Segments)	Antennal flagellum (Segments)	Uropod	Telson	Pleopods	Pereiopods
I	Sessile	0	0	0		Non-articulating join with 6th abdominal somite		
II	Stalked	0	0	0	Simple exopods present	Partially articulating join with 6th abdominal somite		
III		1	3	0	Exopods emerge	Fully articulating join with 6th abdominal somite		
IV		2	3-4	0	Endopods emerge			
V		2	4	1		Shape changes from a fan-like to rectangular		5th pair emerge
VI*		2	5	1			Buds for 3rd and 4th pair emerge, with 5th pair also present on some individuals	
VII**		2-3	6-8	1			Buds for 2nd and 5th pair emerge, already emergent buds for 3rd and 4th pair elongate	
VIII*		3	8	2			3rd and 4th pleopod pairs now biramous, with 5th pair also biramous on some individuals. Buds for 1st pair emerge	
IX*		3-4	9	3			All pleopods now biramous and possess setae. Buds of appendices internae seen on 3rd and 4th pleopods	
X*		4-5	10	4				Chelae on 2nd pair visible
XI**		5-6 or 6-7	14-18	6-8			Appendices internae well developed on 3rd and 4th pleopods	Chelae on 2nd pair larger.
XII*		7-8	15-20	9-12				Chelae on 1st pair visible
XIII*		8-9	29+	14+				
Decapodid		8-9 (v.c.^#^) + 1 (d.c.^#^)	40+	16+				Exopods with natatory setae absent or greatly reduced. Chelae on 2nd pair prominent

*Indicates stages which may have at least 2–3 instars and **indicates stages which may have 3–4 instars. ^#^V.c. refers to the ventral carina and d.c. the dorsal carina of the rostrum.

#### Zoea I

Zoea I larvae of *M. lar* possess a short, straight rostrum which is not toothed. The telson does not possess uropods, is roughly heart-shaped and doesn't articulate, forming a solid join with the sixth abdominal somite. This stage does not possess fully formed walking legs (pereiopods) with the first two pairs present as buds only. The first three maxillipeds pairs are present, the eyes are sessile and located on the anterior half of the cephalothorax. The body is highly transparent and lipid globules can be seen in the foregut and midgut regions (Additional files 1: Figure S1). Most zoea I larvae were not observed to feed immediately after hatch, however some individuals seized and fed on egg custard and biofloc particles. Most individuals were seen feeding on the second day post-hatch.

#### Zoea II

The rostrum remains largely unchanged from zoea I, however the carapace now has pairs of supra-orbital and pterygostomian spines. The most noticeable feature of this stage is the presence of stalked eyes. A join starts forming between the sixth abdominal somite and telson allowing partial articulation, and within the telson, rudimentary uropod exopods may be seen forming which will appear in the next stage (Additional file 2: Figure S2). The antennal flagella are present but not segmented. The first two pereiopods have developed and appear similar to the third maxilliped.

#### Zoea III

The first tooth on the rostrum appears, located immediately behind the eyes on the dorsal carina and the pterygostomian spine develops 2 obvious points. The antennal flagellum becomes divided, and now contains 3 segments. Uropod exopods emerge, and rudimentary uropod endopods can be seen developing inside the telson (Additional file 3: Figure S3). All maxillipeds and pereiopods are better developed in this stage, with the fourth pereiopod appearing as a biramous bud.

#### Zoea IV

The second tooth on the rostrum appears, appearing in front of the first tooth. The fifth pereiopod appears as a uniramous bud, while the fourth pereiopod is no longer a bud and possesses all segments. The uropod endopods now emerge, making the tail fan complete (Additional file 4: Figure S4). This stage is often noticeably pigmented, with chromatophores distributed over various parts of the body.

#### Zoea V

4 and 1 segments are present in the antennal and antennular flagellae respectively. All pereiopods are present now, with the fifth pereiopod becoming fully developed. The second tooth on the dorsal carina of the rostrum remains. The telson has also gradually changed shape, becoming noticeably rectangular from its previous triangular outline (Additional file 5: Figure S5).

#### Zoea VI

The rostrum remains unchanged, with the exception of the appearance of 2 or more setae in front of the second tooth. 5 and 1 segments are present in the antennal and antennular flagellae respectively. Pleopod buds now appear, but usually only for the third and fourth, and occasionally fifth pairs of pleopods (Additional file 6: Figure S6).

#### Zoea VII

This stage is often the first point at which mark-time moulting may be encountered, and variability in morphological development between individuals was observed. The rostral tooth count can vary between 2–3 teeth on the dorsal carina. Upon reaching zoea III, some individuals had their first rostral tooth emerging almost parallel to the supra-orbital spine, thus the tooth was located well behind the eye (termed the post-orbital tooth here). In other individuals, their first rostral tooth emerged immediately behind or parallel to the eye (Additional file 7: Figure S7).

For individuals which had their first rostral tooth well behind the eye, they possessed a total of 3 rostral teeth by the time they moulted to stage VI, whereas others had only 2. Those individuals which had only 2 teeth occasionally developed a protrusion in front of the second dorsal tooth where the next tooth would emerge (see Additional file 11: Figure S11 for an example of this in a Zoea X larva). 6–8 segments are present in the antennal flagellum and 1 segment remains in the antennular flagellum. The pleopod buds have also become more developed, as the third and fourth pairs have elongated and buds for the second and fifth pairs emerged. In individuals which already possessed a fifth pleopod bud, it elongated along with the third and fourth buds.

#### Zoea VIII

The rostral tooth count is usually 3 teeth along the dorsal carina, however some individuals may still possess 2 teeth, as described for zoea VII. 8 and 2 segments are present in the antennal and antennular flagellae respectively. All pleopods which had elongated during the previous stage are biramous now and possess natatory setae. In most individuals, the second pleopod bud elongates, and the first pair of pleopods emerges as a simple bud (Additional file 8: Figure S8).

#### Zoea IX

This larval stage possesses 3–4 teeth along the dorsal carina of the rostrum, and individuals which possessed only 3 teeth did not have a post-orbital rostral tooth. 9 and 3 segments are present in the antennal and antennular flagellae respectively. All pleopods are biramous and possess setae, making their development almost complete. Now that the endopods of the third and fourth pleopods are fully formed, buds of the appendices internae begin to appear along their inner margins (Additional file 9: Figure S9).

#### Zoea X

An additional tooth is added to the rostrum, bringing the tooth count up to 4–5 teeth along the dorsal carina. Individuals which possessed only 3–4 teeth did not have a post-orbital rostral tooth (Additional file 11: Figure S11). 10 and 4 segments are present in the antennal and antennular flagellae respectively. Chelae appear, forming at the ends of the second pair of pereiopods (Additional file 10: Figure S10).

#### Zoea XI

The number of rostral teeth is 6–7, or 5–6 along the dorsal carina. Individuals which possessed 5–6 teeth did not have a post-orbital rostral tooth. 14–18 and 6–8 segments are present in the antennal and antennular flagellae respectively. All pleopods are now fully formed, with complete development of the appendices internae. Chelae on the second pair of pereiopods are now larger, and used by the larva in feeding. It was difficult to ascertain whether chelae on the first pair of pereiopods had developed at this stage on the live specimens examined. The basal segment of the fifth pair of pleopods now begins to develop setae on its rear margin, with 4 present at first appearance (Additional file 12: Figure S12).

#### Zoea XII

The rostral tooth count is 7–8 teeth along the dorsal carina, and individuals which possessed 7 teeth did not have a post-orbital rostral tooth. 15–20 and 9–14 segments are present in the antennal and antennular flagellae respectively. Chelae on the second pair of pereiopods (second chelipeds) further enlarge from the previous stage, and have developed more setae along the pollex and dactylus. Chelae are now evident on the first pair of pereiopods (first chelipeds). The basal segment of the fifth pair of pleopods possesses 8 setae on its rear margin (Additional file 13: Figure S13).

#### Zoea XIII

This was the final zoeal stage observed, with a rostral tooth count of 8–9 teeth along the dorsal carina. Individuals which possessed 8 teeth did not have a post-orbital rostral tooth. >29 segments are present in the antennal flagellum which is twice the length of the scaphocerite, with 14 in the antennular flagellum. The second chelipeds have enlarged and the larva can be seen capturing *Artemia* nauplii with these while feeding. Although smaller in comparison, the first chelipeds are also noticeable when used during feeding. The basal segment of the fifth pair of pleopods possesses 11 setae along its rear margin (Additional file 14: Figure S14). A characteristic habit of this larval stage not noticed in the earlier stages was to sit on the bottom of the tank and walk for short distances using the pereiopod endopods.

#### Decapodid

The rostral tooth count for this stage is 8–9 teeth along the dorsal carina. Individuals which possessed 8 teeth on the dorsal carina did not have a post-orbital rostral tooth, and the ventral carina now bears a single tooth a short distance from the rostral apex. This stage possessed >40 and >16 segments in the antennal and antennular flagellae respectively. The telson now appears triangular from above, with the rear margin coming to a point and resembling that of the adult.

The second chelipeds are now greatly enlarged and are the largest pair of legs. In 2 of the 5 individuals observed, rudimentary natatory exopods were visible after the moult to decapodid had been completed (Additional file 15: Figure S15). This feature, along with the benthic behaviour characteristic of decapodids of other *Macrobrachium* spp. was confirmation that this was indeed the decapodid stage and not yet another zoeal stage. 3 of the 5 decapodids produced were maintained in isolation after having been acclimated to freshwater, and found to moult into the first juvenile after a period of 5 days from metamorphosis. As mass cultures were used for rearing in this study, it was not possible to distinguish the exact number of instars the zoea larvae passed through before metamorphosis.

Two particular stages *viz*. zoea VII and XI, may include more than 2 and up to an estimated 4 instars, as some individuals showed nearly identical morphological features but had increased in size relative to other larvae within the same stage. Any changes noted in morphology were subtle and very minor, e.g. additional setae on the antennal scale, pleopods and pereiopod exopods. This was interpreted as possible evidence of mark time moulting, and in the case of some zoea XI larvae, terminally additive staging. By taking these observations into consideration, the larvae of *M. lar* may moult through a minimum of 22 and maximum of 31 instars before being ready to metamorphose into the decapodid.

## Discussion

Although larvae were morphologically similar to other *Macrobrachium* spp. which display a 'prolonged/normal' (Alekhnovich and Kulesh 2001; Jalihal *et al.*^1993^) development pattern, there were important differences in behaviour, growth and feed preferences.

### Larval behaviour

The larvae displayed a more benthic habit (even in the presence of aeration), unlike *M. rosenbergii* where healthy larvae without aeration remain near the water surface (Valenti *et al.*^2010^). This agrees with Atkinson (1973), who mentions that larvae occupied the upper portion of the water column but were not directly associated with the surface. This may be related to predator avoidance and use of sub-surface currents for larval transport out of coastal waters during dispersal, and could be important for providing feed where larvae are able to easily access it in culture. Cannibalism was not observed during this study although it cannot be conclusively ruled out, whereas this has been documented for *M. rosenbergii* (Valenti *et al.*^2010^). Our observations differ with Nandlal (2010), who reported that *M. lar* larvae did cannibalise.

### Growth and development

Several *Macrobrachium* spp. produce larvae with requirements for oceanic salinity conditions (30–35 ppt). The number of stages described for these ranges from 9 (*M. grandimanus*; Shokita 1985), 10 (*M. equidens*; Ngoc-Ho 1976 and *M. intermedium;* Williamson 1971) and up to 12 (*M.* sp.; Ngoc-Ho 1976 and *M. olfersii*, Dugger and Dobkin 1975), compared to the 13 described here for *M. lar*.

The most obvious trends (Figure 3) are increasing intermoult periods from averages of 4–8 and 12.6 days during zoeal stages I, V and IX respectively, with corresponding shifts in moults into subsequent larval stages. It is well known that *Macrobrachium* spp. exhibit plasticity in the number of instars, morphological development stages and developmental pathways before metamorphosis, as responses to unfavourable environmental conditions, inappropriate nutrition and the presence/absence of settlement cues (Anger 2001).

It is possible that larvae in this study underwent mark-time moulting due to either unfavourable environmental conditions, inappropriate nutrition or both, and further investigation is required to determine this. Nevertheless, it appears that conditions were sufficient to allow 5 larvae to metamorphose into decapodids. Evidence of mark time moulting has also been reported in other *Macrobrachium* spp., particularly those which inhabit marine or partly-marine conditions as adults, *viz. M. equidens* (Ngoc-Ho 1976), *M. rosenbergii* (Gomez Diaz and Kasahara 1987; Valenti *et al.*^2010^) and *M. vollenhovenii* (Müller *et al.*^2003^).

Observations of inherent developmental plasticity have been related to the wide marine dispersal capacity of some species (Shokita 1985), and is an important factor to consider in developing commercial hatchery operations, as extended larval development duration increases operating costs. Other *Macrobrachium* spp. which share a wide Indo-Pacific distribution with *M. lar* and exhibit developmental variability include *M. grandimanus* (Shokita 1985) and *M. equidens* (Ngoc-Ho 1976). Studies of the population structure of *M. lar* using mitochondrial DNA markers in Japan (Imai *et al.*^2007^) and in Pacific Island countries (Mather *et al.*^2006^; Nandlal 2010) have shown high genetic diversity over large geographic scales. This implies substantial gene flow between widely separated habitats, and indicates long-lived pelagic larvae able to colonise habitats far removed from their place of hatch.

### Feeds and feeding

The observation that the larvae may have different feed preferences to other *Macrobrachium* spp. requires further investigation. The larvae of most *Macrobrachium* spp. are omnivorous, with carnivorous tendencies. This has been demonstrated for *M. rosenbergii* up to zoea VII, after which they become more omnivorous (Dhont *et al.*^2010^). Reasons stated for this include the larvae remaining primitive during early development, with only partially developed systems for digestion, sight and chemoreception. The gut remains poorly developed until larval stages V and VI, resulting in a low digestive capacity and hence the early stages are reliant on highly digestible live feeds (*eg.* zooplankton), which provide exogenous prey enzymes to begin the proper processes of digestion (Dhont *et al.*^2010^).

The primary feed used for most other species including *M. rosenbergii* (Ling 1961, 1962; Uno and Kwon 1969), *M. vollenhovenii* (Willführ-Nast *et al.*^1993^), *M. carcinus* (Choudhury 1971b, 1971c), *M. novaehollandiae* (Greenwood *et al.*^1976^), *M. americanum* (Monaco 1975; Holtschmit and Pfeiler 1984), *M. equidens* (Ngoc-Ho 1976) and *M. acanthurus* (Choudhury 1970, 1971a) is the nauplii of *Artemia* spp. If larval *M. lar* are proven to show a preference for plant-based feeds, this may imply lower feed-associated costs as they are generally cheaper to obtain.

Previous studies which reared *M. lar* had used *Artemia* nauplii as the staple feed with varying results, and all failed to reach the decapodid stage (Kubota 1972; Atkinson 1973, 1977; Nandlal 2010). Supplementary feeds utilised included ox liver particles (Nandlal 2010), Melon Fly *Bactrocera* (*Dacus*) *cucurbitae* larvae along with a prepared feed incorporating shrimp meal (20%; Atkinson 1973, 1977).

The production of decapodids in this study may be partly attributed to a more suitable larval diet. The specific feeds which may have met larval nutritional needs were the prepared custard feeds and biofloc. It is likely that the custard feeds supplied dietary energy requirements during the later zoeal stages when they were easier to metabolise, with biofloc being important earlier during development. Avnimelech (2009) mentions that suspended biofloc is eaten and contributes significantly to the protein requirements of species reared in Biofloc Technology systems including Tilapia, various Carp and the marine shrimp *Litopenaeus vannamei* and *Penaeus monodon*.

### Larval rearing and survival

The overall survival rate from hatch till metamorphosis was very low (0.08%), similar to likely rates in the wild of <0.1% (Bagenal 1967; Jennings *et al.*^2006^), and dependant on temperature, salinity, food availability and development/settlement cues (Willführ-Nast *et al.*^1993^; Anger 2001). In crustacean species for which larviculture techniques are being developed, larval survival rates in early trials are not much better than those inferred for wild larvae. As an example, initial research on the Mud Crab *Scylla* sp. in Indonesia produced survival rates till metamorphosis of 0.07–0.19 and 0.5–3.2% (Cholik 1999).

Survival rates reported for other *Macrobrachium* spp. have been comparatively low during initial attempts, but have improved with continued refinement of culture techniques. Perhaps the best example of this is *M. rosenbergii*. When decapodids for this species were first produced, the survival rate till metamorphosis was 16–17% (Ling 1961, 1962). Today, survival rates are 40–50% in flow-through hatchery systems, 60–80% in Thai backyard hatcheries and 60–80% in experimental and commercial recirculation systems, with development durations of 29–35 days (Valenti *et al.*^2010^). It can thus be expected that there will be room for improvement in *M. lar* larviculture performance as a result of further research.

Survival rates reported for other *Macrobrachium* spp. assessed for culture potential are varied. Survival till metamorphosis was 12% for *M. vollenhovenii* (Willführ-Nast *et al.*^1993^), 21% and 2.5% for *M. acanthurus* and *M. carcinus* respectively (Dobkin *et al.*^1974^), 9% for *M. acanthurus* (Choudhury 1971a), >90% for *M. amazonicum* (Anger *et al.*^2009^), 20% for *M. americanum* (Holtschmit and Pfeiler 1984) and ~59% for *M. nipponense* (MacLean and Brown 1991). It is difficult to compare these rates with those of *M. lar* in this study, as some of the species do not have larvae which require fully marine conditions for development.

Those species which have a requirement for >20 ppt include *M. vollenhovenii* (16–24 ppt), *M. acanthurus* (<20 ppt) and *M. americanum* (20–30 ppt for early larval stages only; Choudhury 1971a; Dobkin *et al.*^1974^; Holtschmit and Pfeiler 1984 and Willführ-Nast *et al.*^1993^). Although a more detailed discussion of the salinity requirements for *M. lar* is provided in Lal *et al.* (2012), salinity tolerance investigations indicated that survival and development of newly-emerged larvae was highest in entirely fresh or slightly brackish water, increasing to full-strength seawater by the mid-point of larval development. Past this point, salinities >30 ppt were critical for larvae to progress past stages VII and VIII (Lal *et al.*^2012^). Larvae were also unable to survive in freshwater beyond a period of 4 days, confirming that this species has a truly oceanic larval dispersal phase (Kubota 1972; Maciolek 1972; Mather *et al.*^2006^; Nandlal 2010).

Another consideration is that survival for extended periods may be genetically 'hard wired' due to the prolonged larval dispersal phase. Despite this, optimising culture methods can be expected to shorten development time, as the first decapodid was produced on day 77 of culture here, whereas Atkinson's (1973, 1977) study reached zoea XI on day 89 before all larvae died.

### Culture system

There has been considerable debate over whether greenwater or clearwater culture systems are better suited for rearing larvae of *Macrobrachium* spp. While both systems have their merits, clearwater systems have been proven easier to manage (Valenti *et al.*^2010^). During this study, the propagation of biofloc in the LRTs was likely to have provided adequate nutrition to some larvae, and responsible at least in part for enabling completion of development and metamorphosis of decapodids. While it remains unclear if larvae derived benefits from feeding directly on the microalgal particles bound up in the biofloc or on biofloc-associated biota, if the simple greenwater system used here can be adapted for mass seed production, the potential to realise cost, time and labour savings by using non-monospecific microalgal cultures is substantial, especially in resource-poor regions where hatchery production of *M. lar* is a priority.

Several studies have examined the role that microalgae play in the larviculture of *Macrobrachium* spp.. Lober and Zeng (2009) found higher survival and shorter development duration in *M. rosenbergii* reared at higher vs. lower microalgal concentrations, while reduced ammonia levels (Cohen *et al.*^1976^) and enhanced survival and metamorphosis rates were seen when larvae were cultured with 7 species of unicellular algae (Manzi and Maddox 1977; Manzi *et al.*^1977^).

Although larval *Macrobrachium* spp. are known to be visual, particulate feeders (Atkinson 1973, 1977), and do not feed directly on algal cells except via accidental ingestion in negligible amounts (Cohen *et al.*^1976^), the benefits of microalgal enrichment of *Artemia* nauplii have been well documented (Dhont *et al.*^2010^; Valenti *et al.*^2010^). Cohen *et al.* (1976) report the presence of algae facilitate growth only indirectly by removing toxic material *e.g.* ammonia, however when considering the incorporation of microalgal cells into biofloc particles in the current study, they would offer similar benefits as enriched *Artemia*. Further mention is made that when incorporating microalgae, the balance of the ecological system in the LRT is more complicated, as more trophic levels exist and less control can be exercised over the whole system. Contrary to this, *M. rosenbergii* has been reared using no intensive hatchery techniques in a greenwater system operated completely without water exchange (Cheah and Ang 1979). LRTs were topped up with greenwater to counter evaporative losses under two salinity regimes of 6–8 ppt and 12–14 ppt. Results showed no significant difference in survival rates to the decapodid of 39.6% and 36.9% for the two regimes respectively.

## Conclusions

Based on the results of this study, commercial-scale hatchery operations for *M. lar* require further research into improvement of larval survival and reduction in development duration to ensure feasibility. Nonetheless, our research provides a record of the first ever complete larval development of *M. lar* with accompanied morphological descriptions, both of which are key tools for successful larviculture for it and potentially other related species.

## Electronic supplementary material

Additional file 1: Figure S1: Zoea I. Lateral view (a), dorso-lateral view of carapace showing sessile eyes (b) and non-articulating telson with sixth abdominal somite join (c). (PDF 785 KB)

Additional file 2: Figure S2: Zoea II. Dorsal view (a) and lateral view (b). Rudimentary uropod exopod development within telson (arrows) (c), formation of join between telson and sixth abdominal somite (arrow) (d) and supra-orbital spine (s.o.s.) (e). (PDF 2 MB)

Additional file 3: Figure S3: Zoea III. Dorsal view (a) and lateral view (b) of larva. Antennal flagellum containing three segments (c), emergent uropod exopods and rudimentary uropod endopods visible within the telson (d). First rostral tooth on the dorsal carina (e). (PDF 2 MB)

Additional file 4: Figure S4: Zoea IV. Dorsal view (a) and lateral view (b). Second rostral tooth on the dorsal carina (c), uniramous buds which are the undeveloped fifth pereiopods (arrows) (d) and complete tail fan development with the emergence of the uropod endopods (e). (PDF 1 MB)

Additional file 5: Figure S5: Zoea V. Dorsal view (a) and lateral view (b). Telson almost rectangular (c), two teeth still present on the dorsal carina (d) and the fully developed fifth pereiopod (e). (PDF 2 MB)

Additional file 6: Figure S6: Zoea VI. Lateral view (a). Emergent buds for the third, fourth and fifth pairs of pleopods (b). Two setae present in front of the second rostral tooth (c). (PDF 1 MB)

Additional file 7: Figure S7: Zoea VII. Lateral view (a). Elongated third and fourth pleopod bud pairs (b) and 6 – 8 segments in the antennal flagellum (c). Two setae still present in front of the second rostral tooth (d). (PDF 1 MB)

Additional file 8: Figure S8: Zoea VIII. Lateral view (a). Third rostral tooth on the dorsal carina (b) and further pleopod development (c). 2 segments present in the antennular flagellum (arrows; d) and 8 segments in the antennal flagellum (e). (PDF 1 MB)

Additional file 9: Figure S9: Zoea IX. Lateral view of larva (a). 3 segments present in the antennular flagellum (arrow; b) and 9 segments in the antennal flagellum (c). Fourth rostral tooth on the dorsal carina (obscured by eye but position indicated by arrow; d) and all pleopods now biramous with setae (e). Buds of the appendices internae are visible developing along the inner margins of the third and fourth pleopod pair endopods (arrows). (PDF 1 MB)

Additional file 10: Figure S10: Zoea X. Lateral view (a). Four segments present in the antennular flagellum (arrows; b) and fifth rostral tooth present on the dorsal carina (c). Ten segments in the antennal flagellum (d) and rudimentary chelae present on the second pair of pereiopods (arrow; e). (PDF 972 KB)

Additional file 11: Figure S11: Zoea X showing variable rostral dentition. Individual with a post-orbital tooth (a) and an individual without a post-orbital tooth displaying a protrusion of the carapace (arrow; b). (PDF 1 MB)

Additional file 12: Figure S12: Zoea XI. Lateral view (a). Chelae present on the second pair of pereiopods are now larger (arrow; b) and appendix interna development is complete on all pleopods (arrows; c). This individual has 5 teeth on the dorsal carina (d). 14 – 18 segments present in the antennal flagellum (e). (PDF 1 MB)

Additional file 13: Figure S13: Zoea XII. Lateral view (a). This individual has 8 teeth on the dorsal carina (b). 9 segments present in the antennular flagellum (c) and ~20 segments in the antennal flagellum (d). 8 setae present on the rear margin of the basal segment of the fifth pair of pleopods (e). (PDF 1 MB)

Additional file 14: Figure S14: Zoea XIII. Lateral view (a). This individual has 9 teeth on the dorsal carina (b). Chelae present on the second pair of pereiopods are now further enlarged (c) and 11 setae present on the rear margin of the basal segment of the fifth pair of pleopods (d). 14 segments present in the antennular flagellum (e). (PDF 1 MB)

Additional file 15: Figure S15: Decapodid. Lateral view (a). This individual has 8 teeth on the dorsal carina (b). The first rostral tooth on the ventral carina (arrow; c) and greatly enlarged second pair of pereiopods and chelae (d). Rudimentary natatory pereiopod exopodites (arrows; e) and triangular telson (f). 16+ segments present in the antennular flagellum (g). (PDF 1 MB)

## Abbreviations

ACIAR: Australian Centre for International Agricultural Research
DO_2_: Dissolved oxygen
FRP: Fibre-reinforced plastic
LRT: Larval rearing tank
mg/L: Milligrams per litre
mm: Millimetres
ppt: Parts per thousand.
