# Supplementary figures and images for: Complete larval development of the Monkey River Prawn Macrobrachium lar (Palaemonidae) using a novel greenwater technique
Source: Springerplus. 2014 Sep 30;3:568. doi: 10.1186/2193-1801-3-568 (PMC4190279; doi:10.1186/2193-1801-3-568)

a

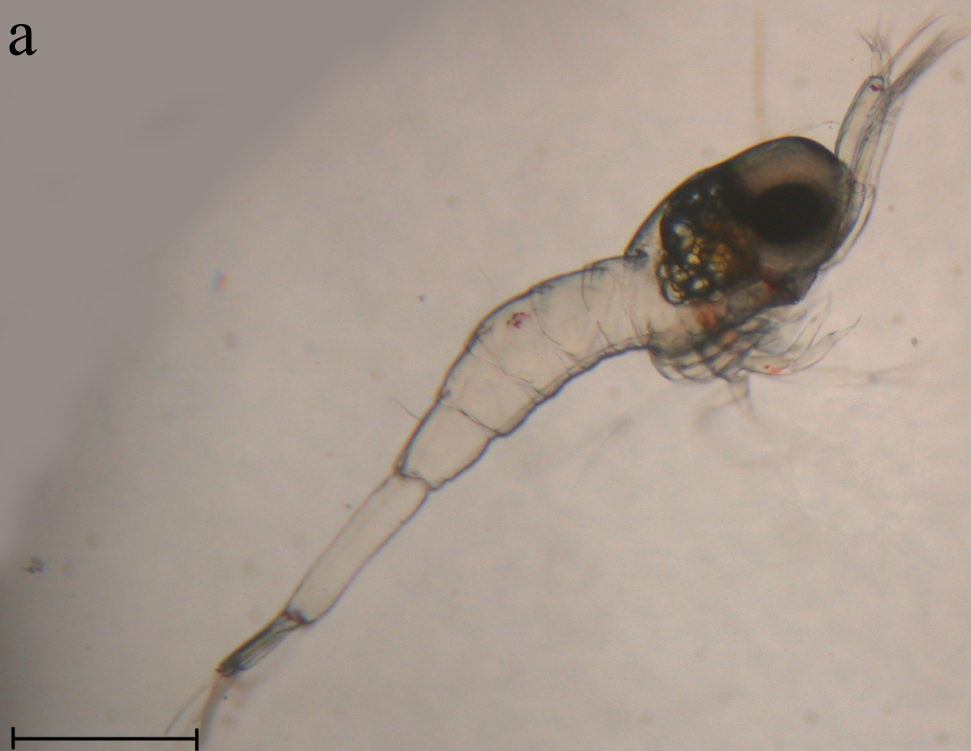

0.5 mm

b

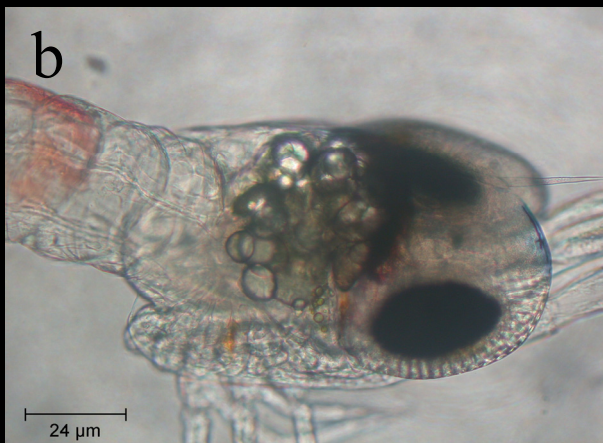

c

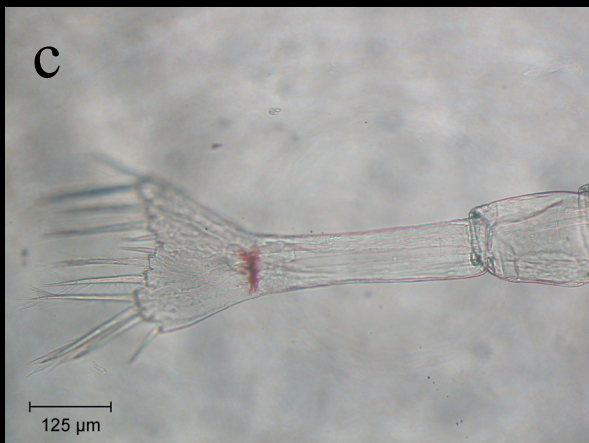

Supplement: Supplementary file 1 — Additional file 1: Figure S1: Zoea I. Lateral view (a), dorso-lateral view of carapace showing sessile eyes (b) and non-articulating telson with sixth abdominal somite join (c). (PDF 785 KB) [file 40064_2014_1266_MOESM1_ESM.pdf]

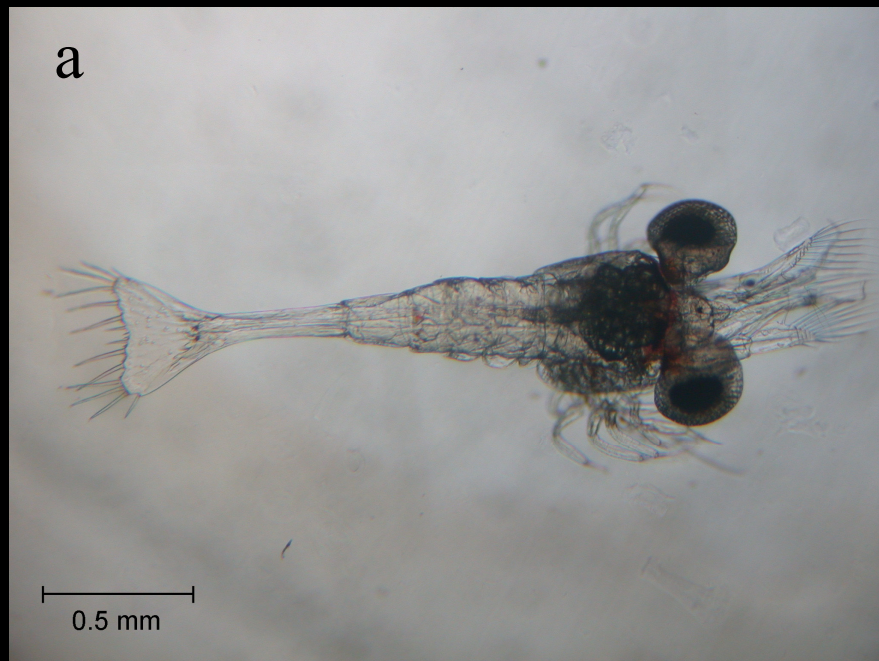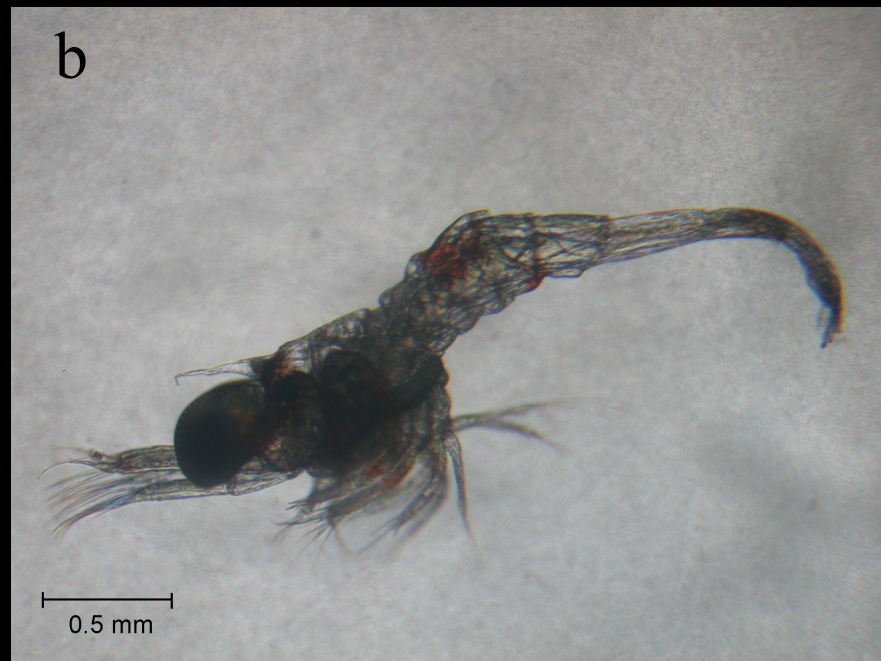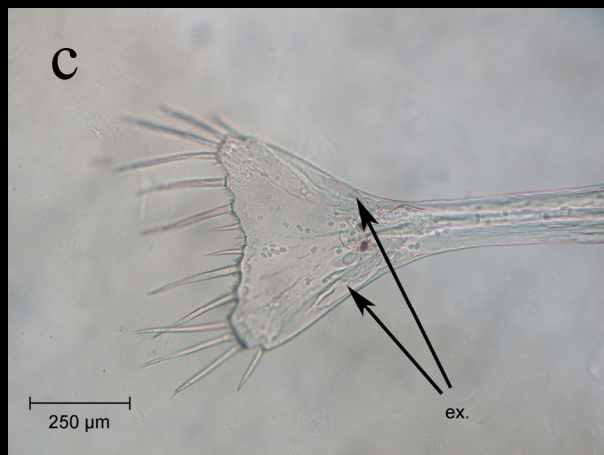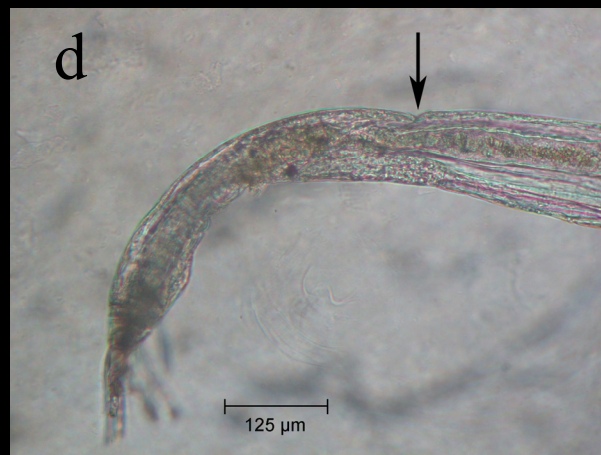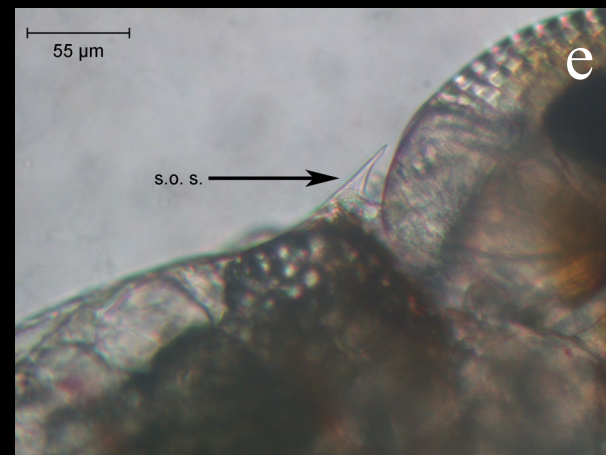

Supplement: Supplementary file 2 — Additional file 2: Figure S2: Zoea II. Dorsal view (a) and lateral view (b). Rudimentary uropod exopod development within telson (arrows) (c), formation of join between telson and sixth abdominal somite (arrow) (d) and supra-orbital spine (s.o.s.) (e). (PDF 2 MB) [file 40064_2014_1266_MOESM2_ESM.pdf]

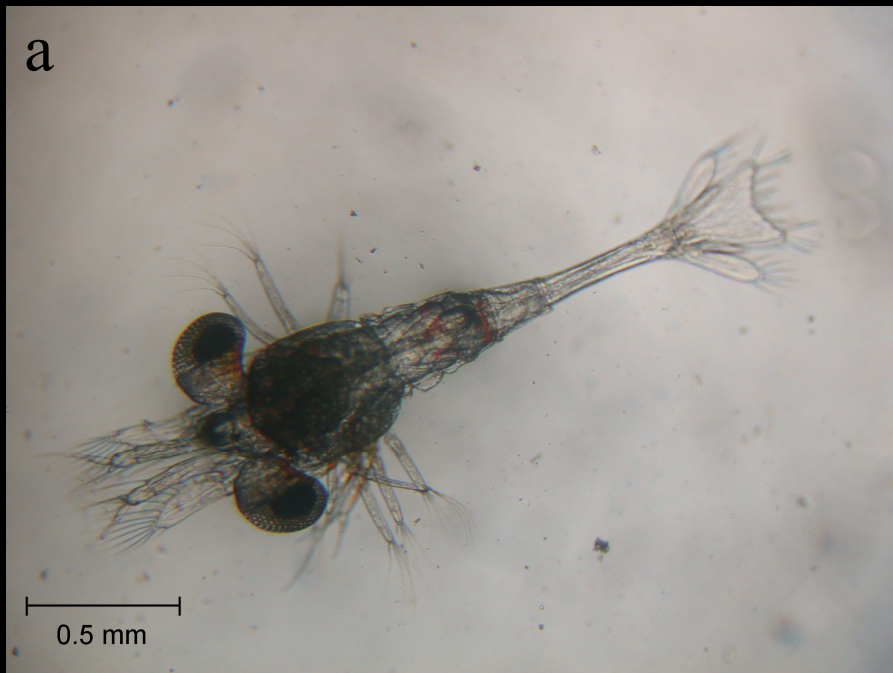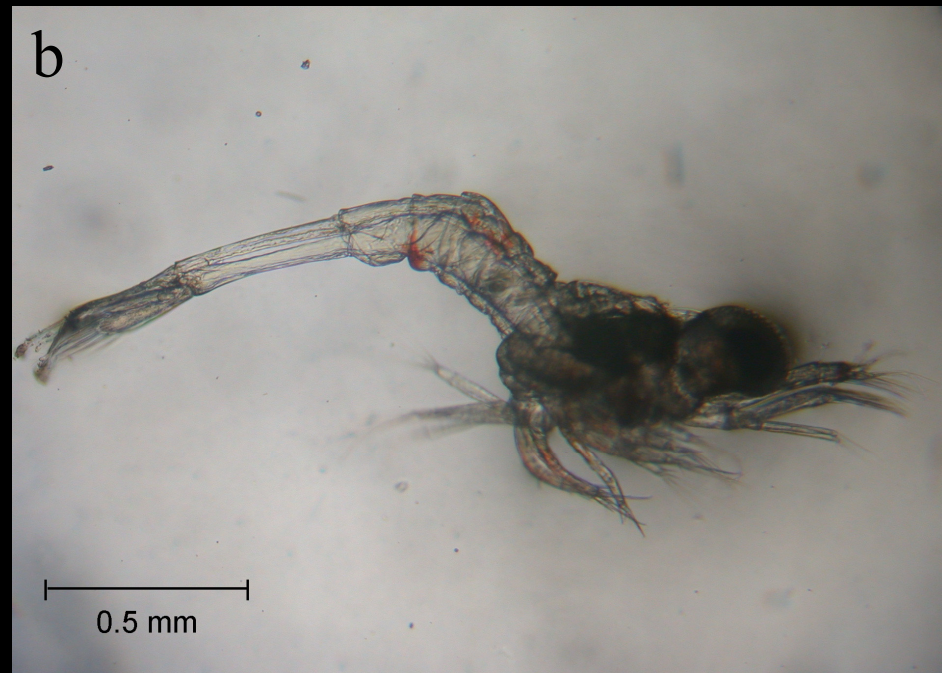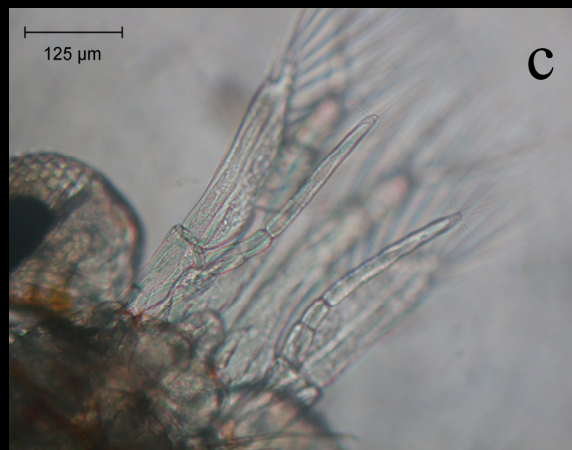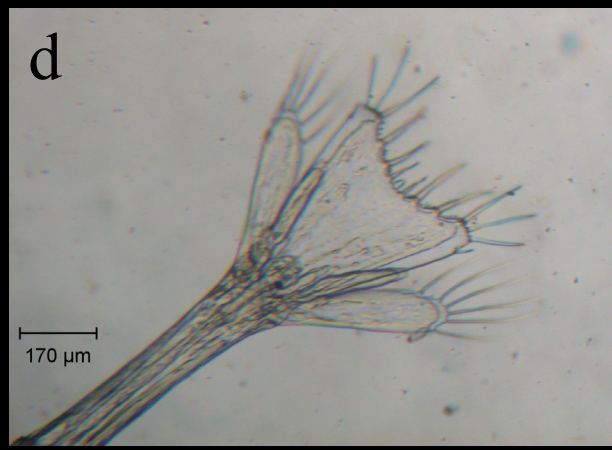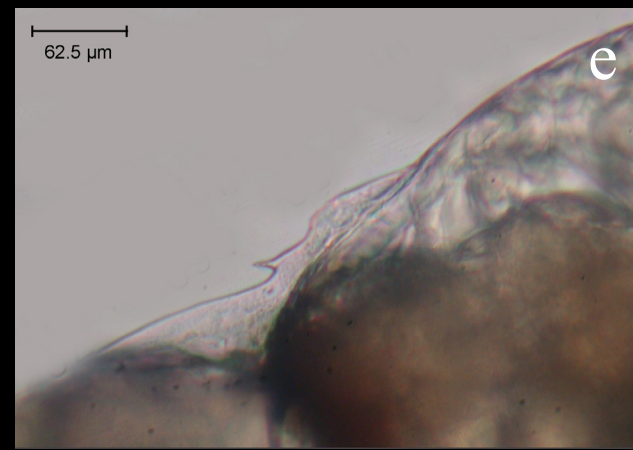

Supplement: Supplementary file 3 — Additional file 3: Figure S3: Zoea III. Dorsal view (a) and lateral view (b) of larva. Antennal flagellum containing three segments (c), emergent uropod exopods and rudimentary uropod endopods visible within the telson (d). First rostral tooth on the dorsal carina (e). (PDF 2 MB) [file 40064_2014_1266_MOESM3_ESM.pdf]

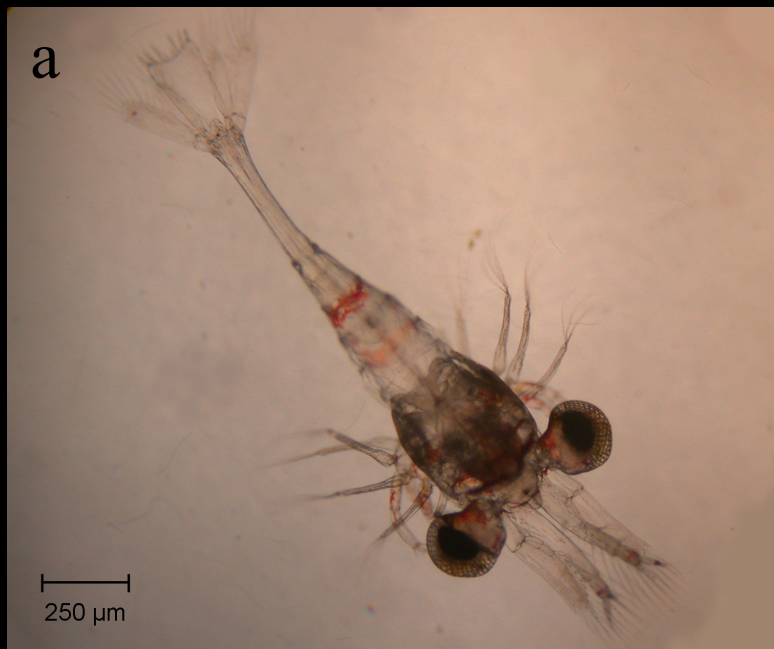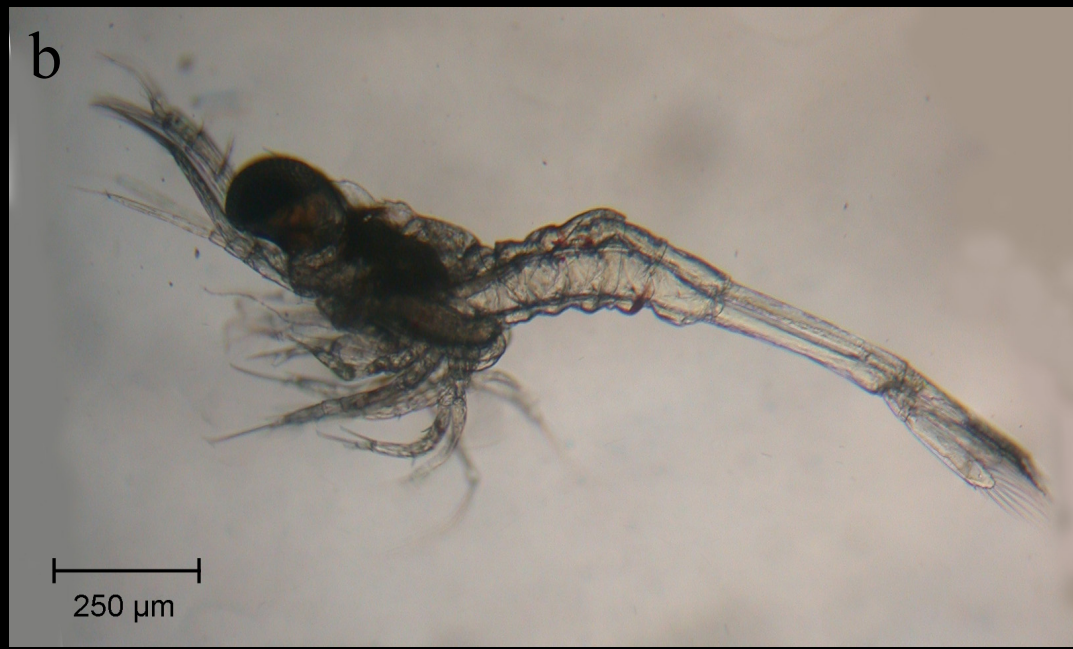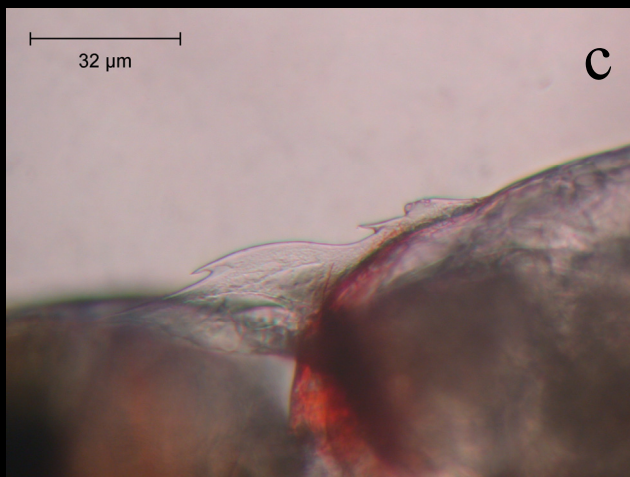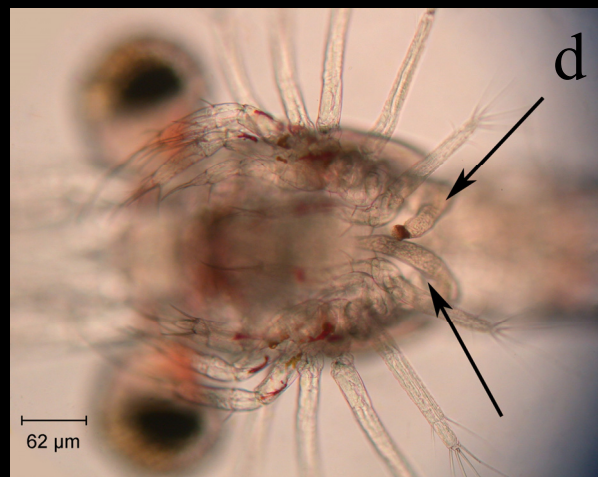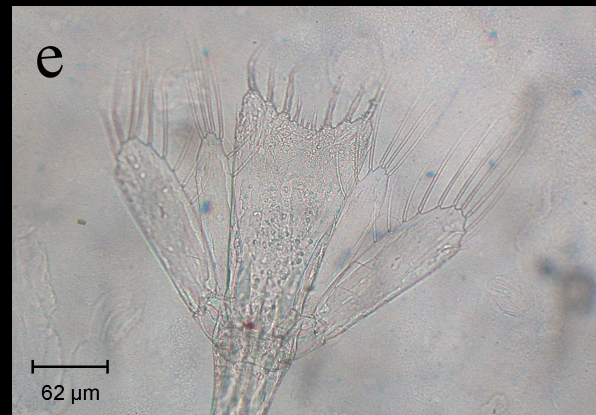

Supplement: Supplementary file 4 — Additional file 4: Figure S4: Zoea IV. Dorsal view (a) and lateral view (b). Second rostral tooth on the dorsal carina (c), uniramous buds which are the undeveloped fifth pereiopods (arrows) (d) and complete tail fan development with the emergence of the uropod endopods (e). (PDF 1 MB) [file 40064_2014_1266_MOESM4_ESM.pdf]

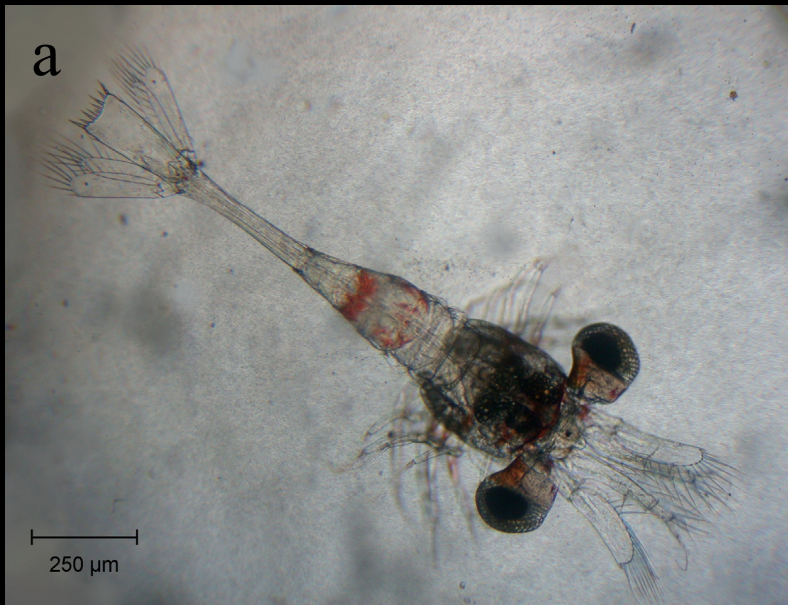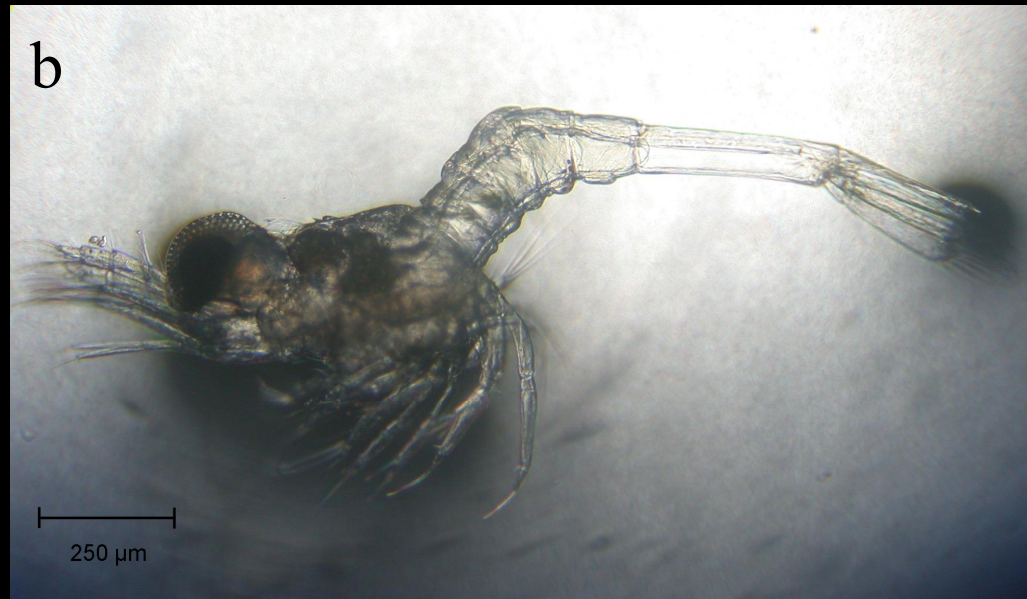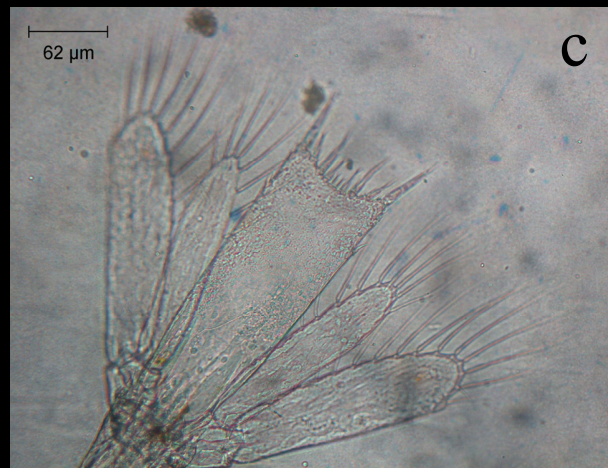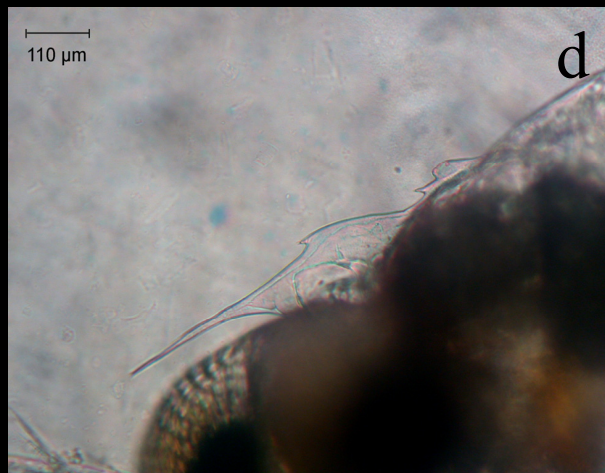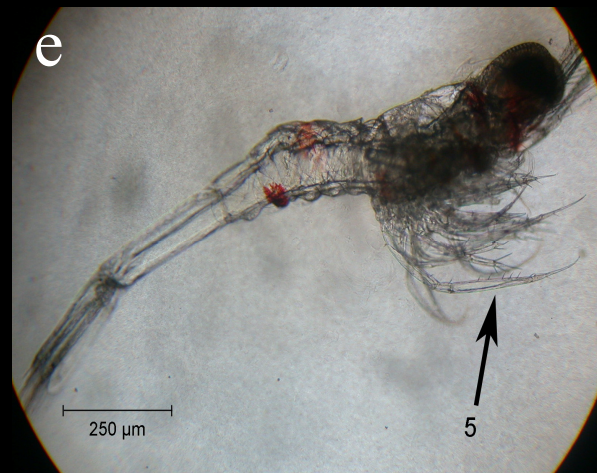

Supplement: Supplementary file 5 — Additional file 5: Figure S5: Zoea V. Dorsal view (a) and lateral view (b). Telson almost rectangular (c), two teeth still present on the dorsal carina (d) and the fully developed fifth pereiopod (e). (PDF 2 MB) [file 40064_2014_1266_MOESM5_ESM.pdf]

a

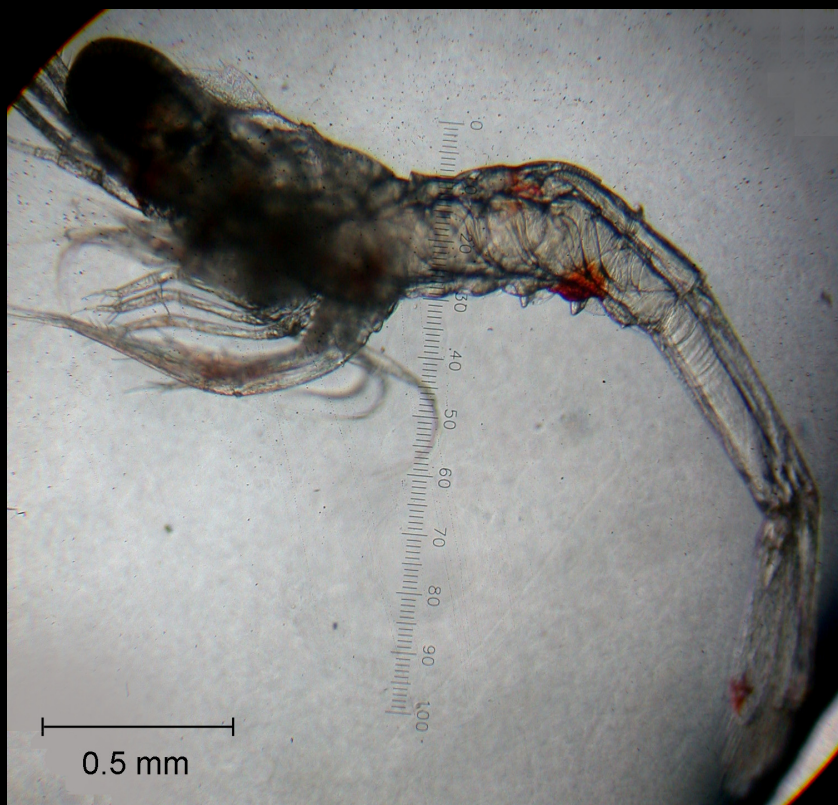

b

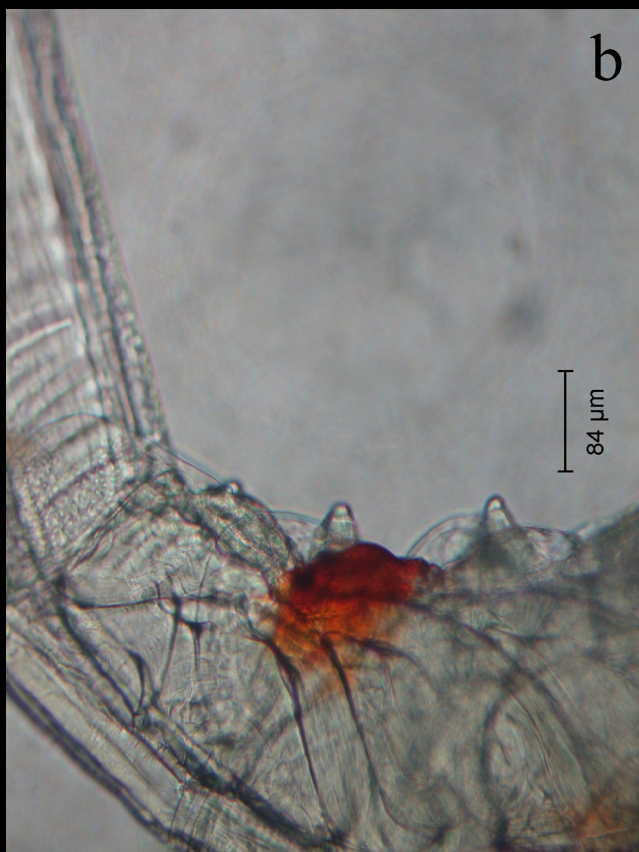

c

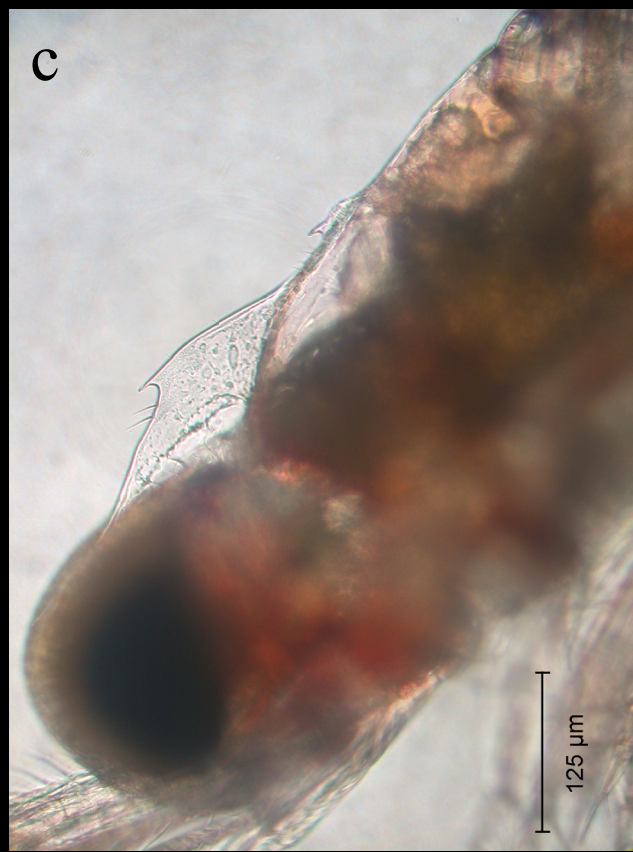

Supplement: Supplementary file 6 — Additional file 6: Figure S6: Zoea VI. Lateral view (a). Emergent buds for the third, fourth and fifth pairs of pleopods (b). Two setae present in front of the second rostral tooth (c). (PDF 1 MB) [file 40064_2014_1266_MOESM6_ESM.pdf]

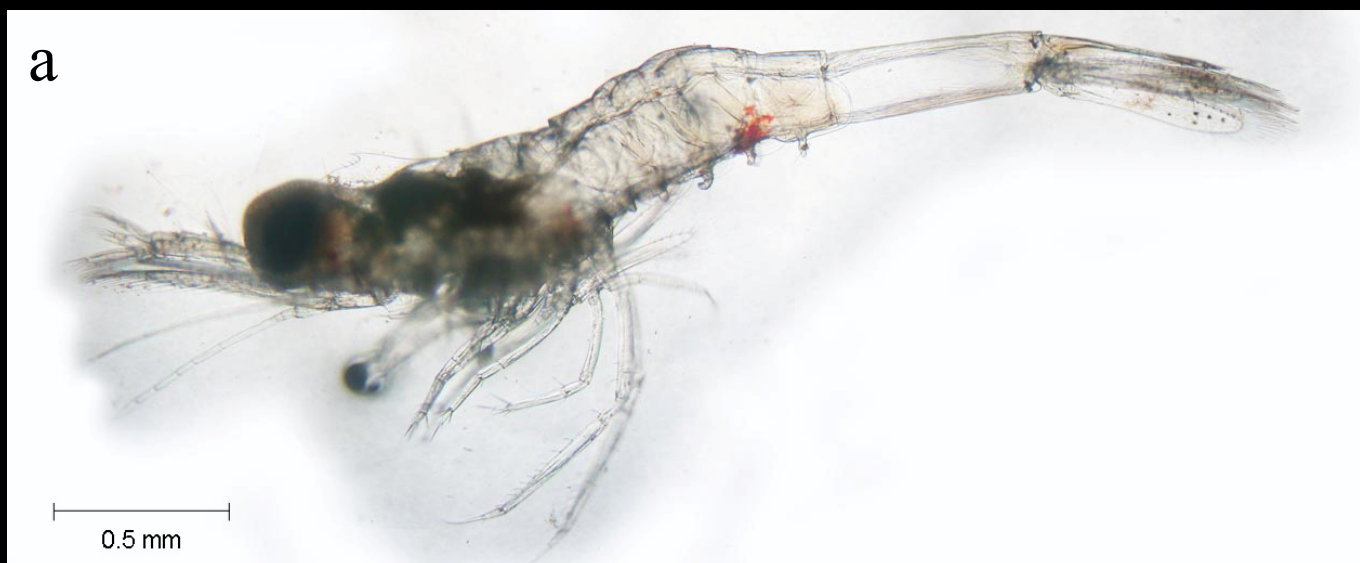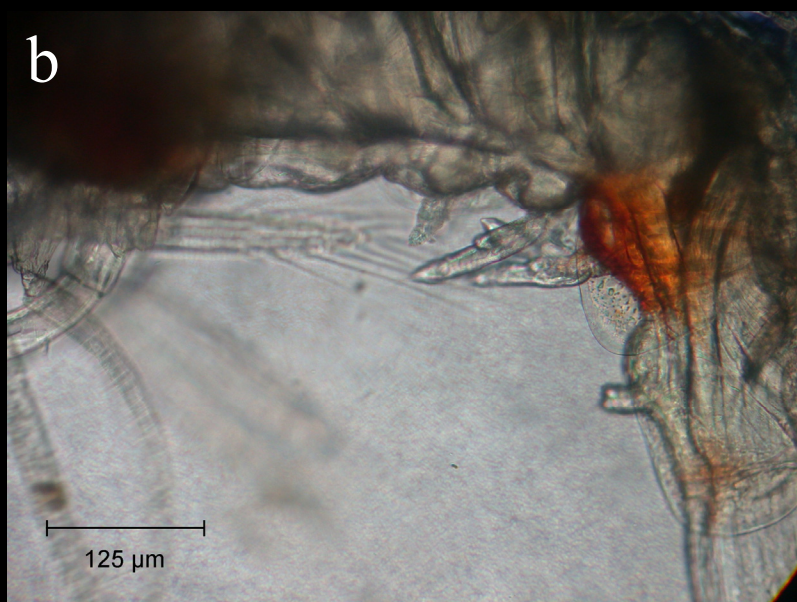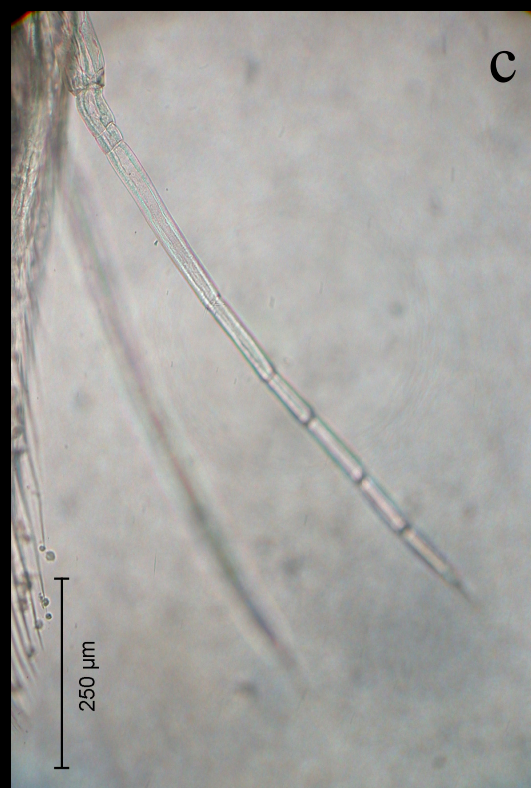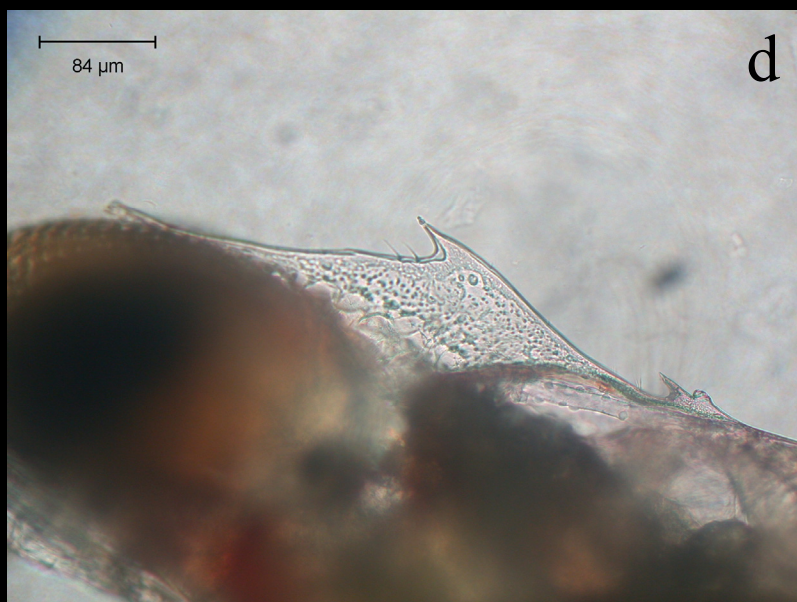

Supplement: Supplementary file 7 — Additional file 7: Figure S7: Zoea VII. Lateral view (a). Elongated third and fourth pleopod bud pairs (b) and 6 – 8 segments in the antennal flagellum (c). Two setae still present in front of the second rostral tooth (d). (PDF 1 MB) [file 40064_2014_1266_MOESM7_ESM.pdf]

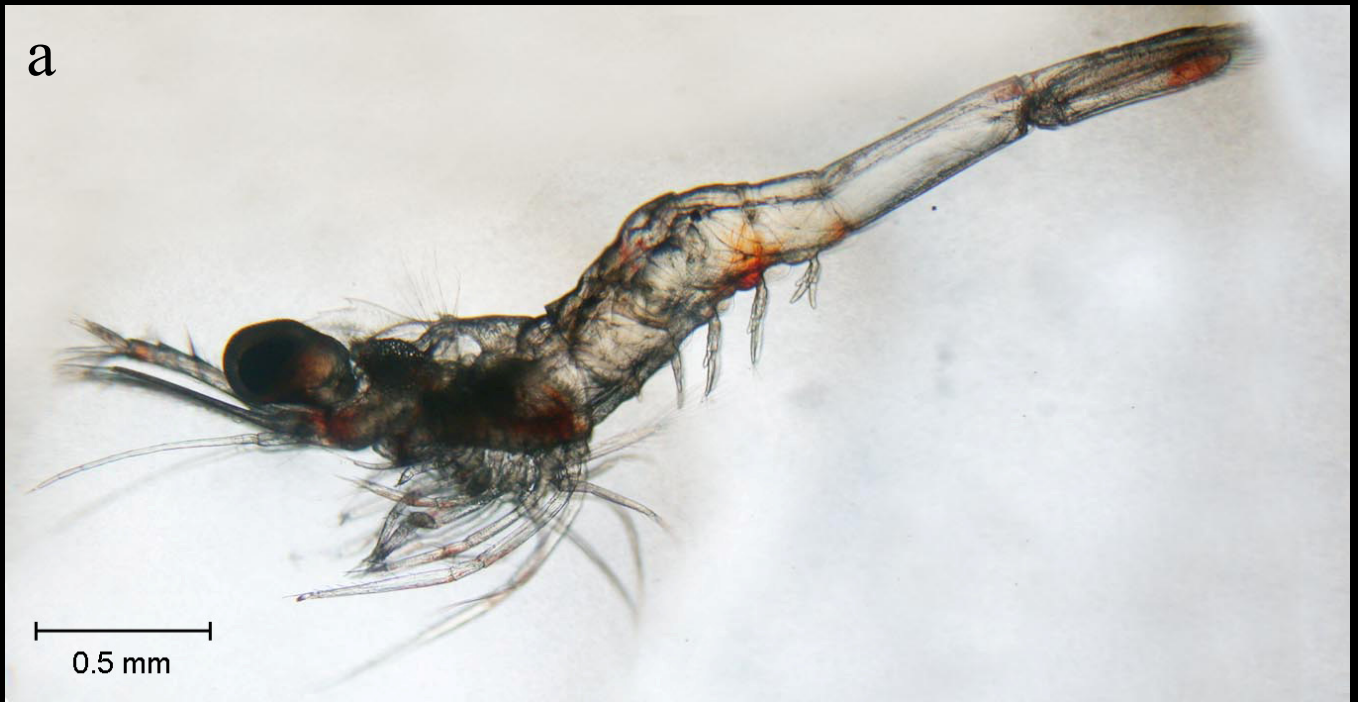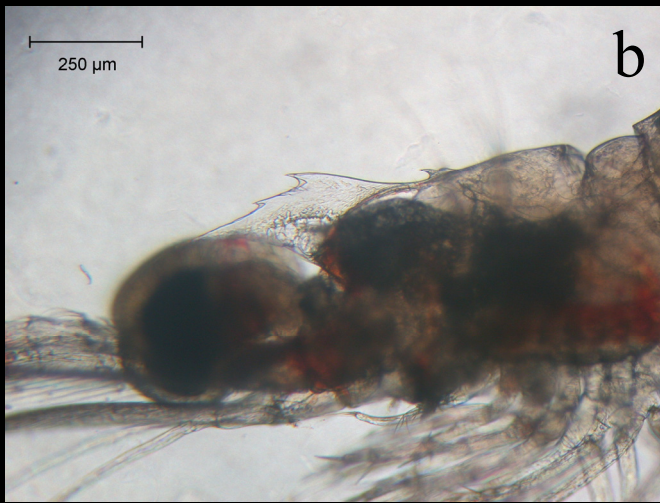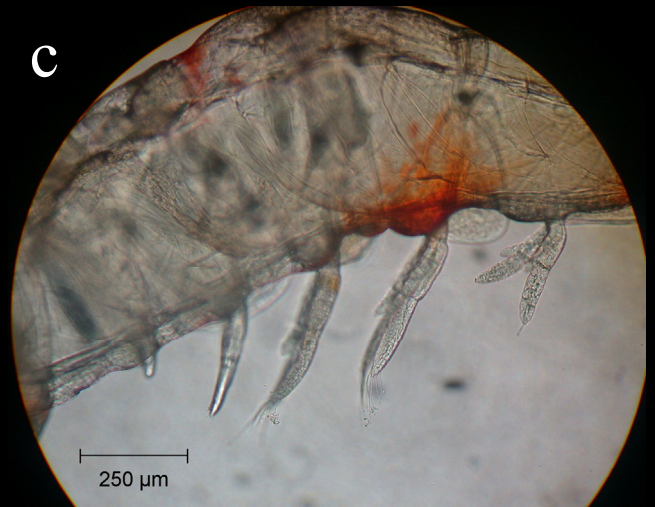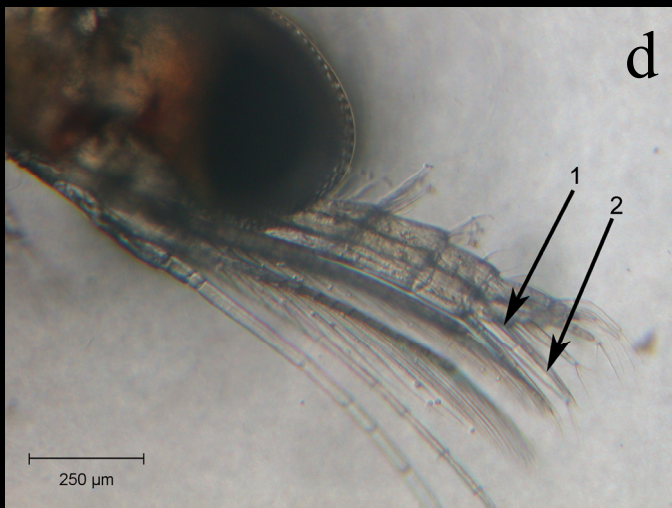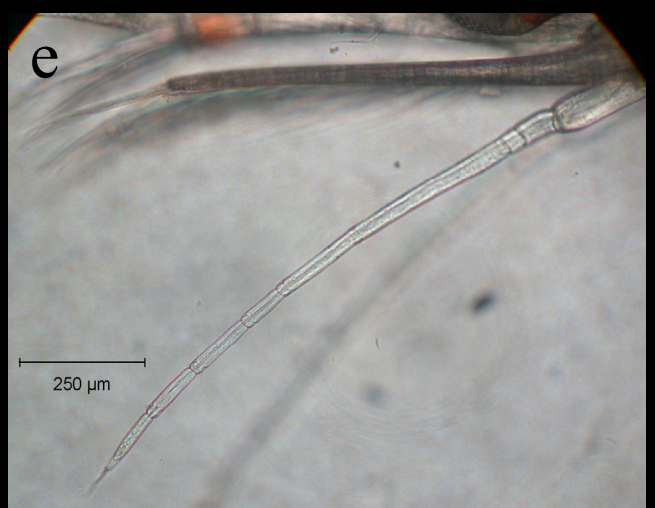

Supplement: Supplementary file 8 — Additional file 8: Figure S8: Zoea VIII. Lateral view (a). Third rostral tooth on the dorsal carina (b) and further pleopod development (c). 2 segments present in the antennular flagellum (arrows; d) and 8 segments in the antennal flagellum (e). (PDF 1 MB) [file 40064_2014_1266_MOESM8_ESM.pdf]

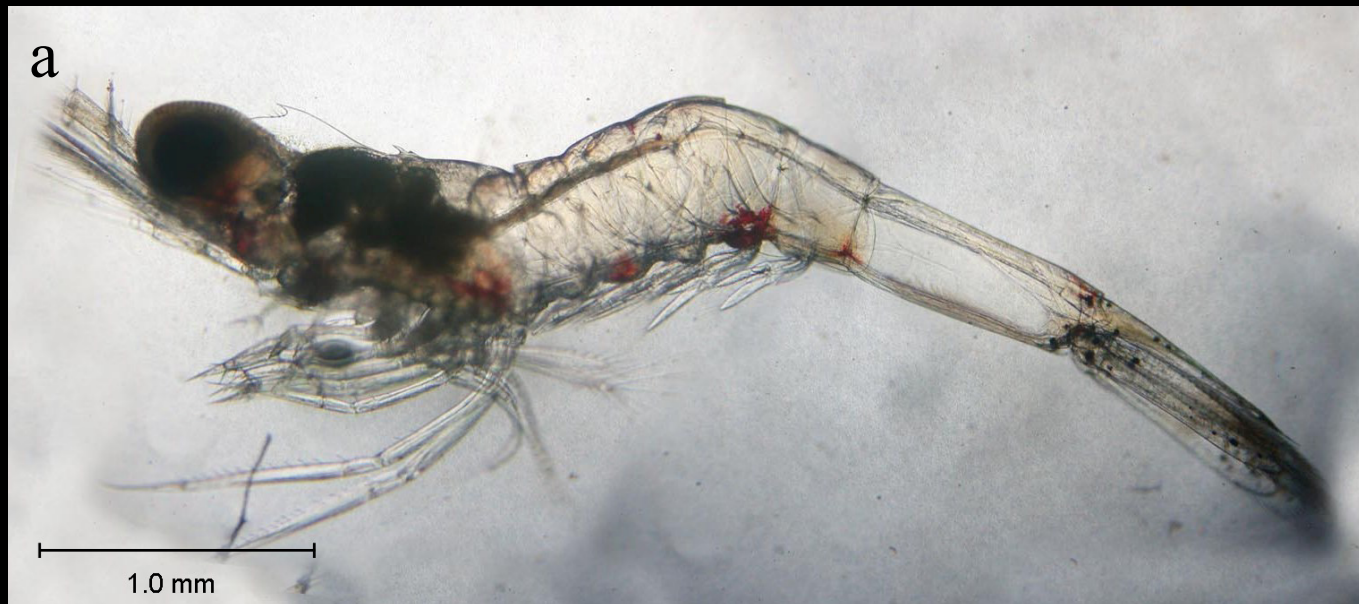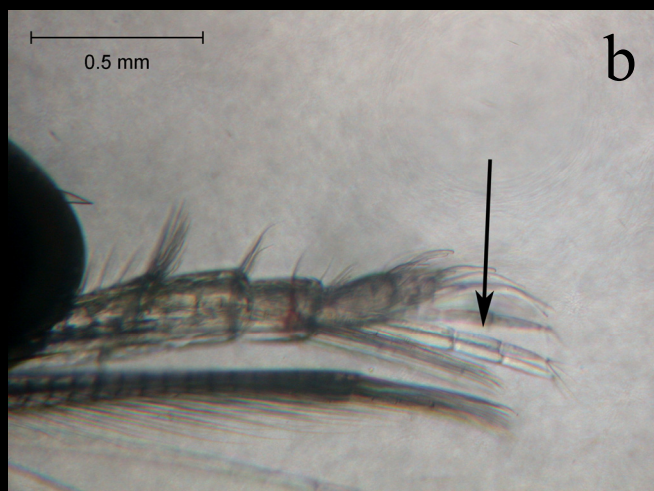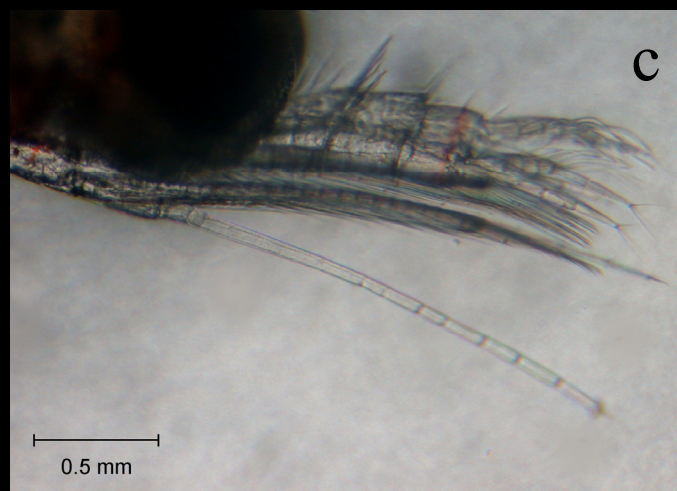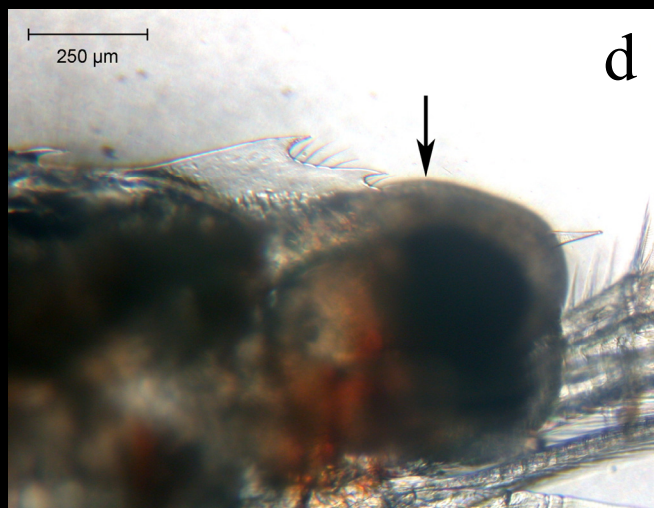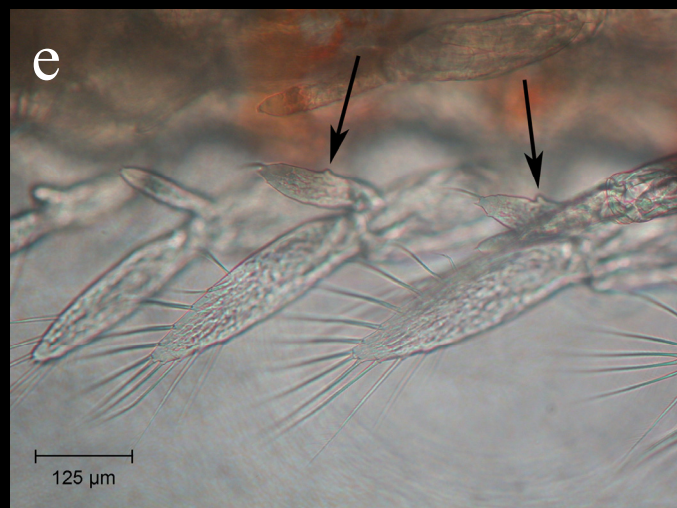

Supplement: Supplementary file 9 — Additional file 9: Figure S9: Zoea IX. Lateral view of larva (a). 3 segments present in the antennular flagellum (arrow; b) and 9 segments in the antennal flagellum (c). Fourth rostral tooth on the dorsal carina (obscured by eye but position indicated by arrow; d) and all pleopods now biramous with setae (e). Buds of the appendices internae are visible developing along the inner margins of the third and fourth pleopod pair endopods (arrows). (PDF 1 MB) [file 40064_2014_1266_MOESM9_ESM.pdf]

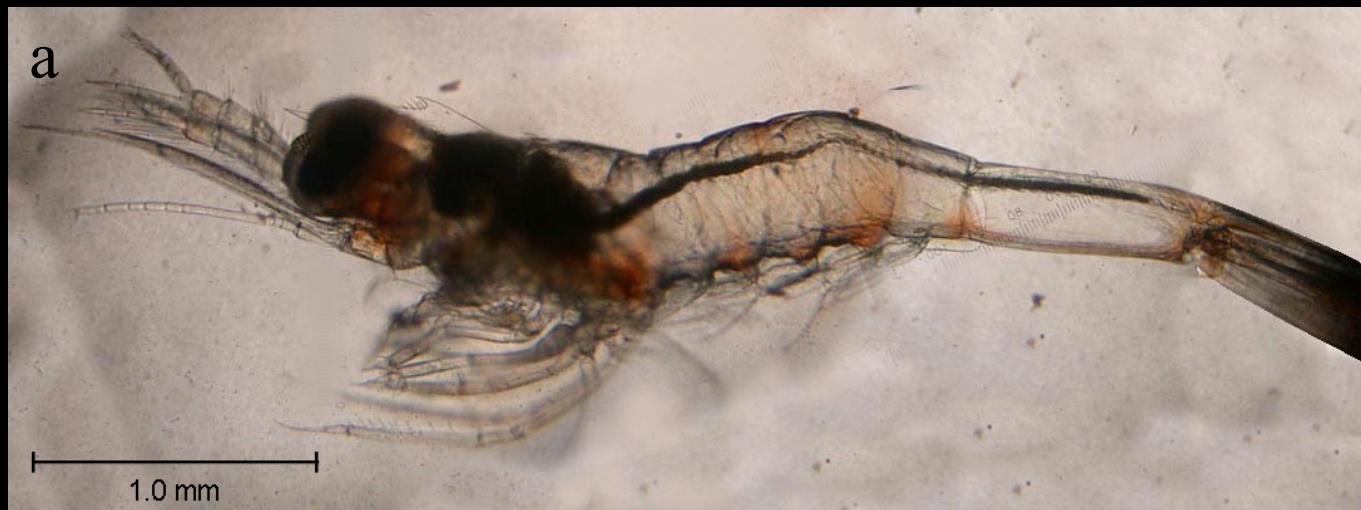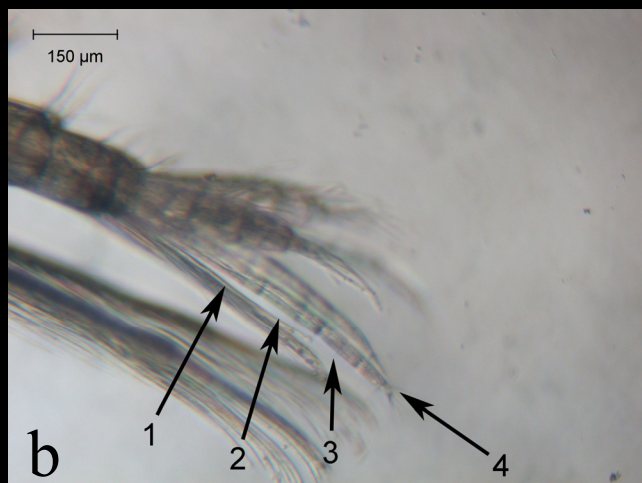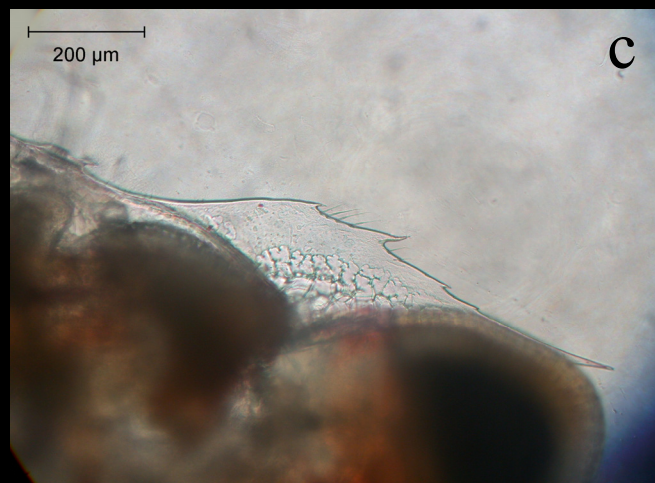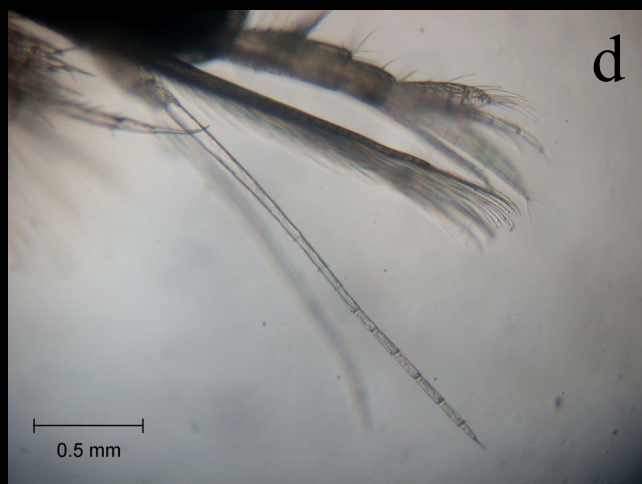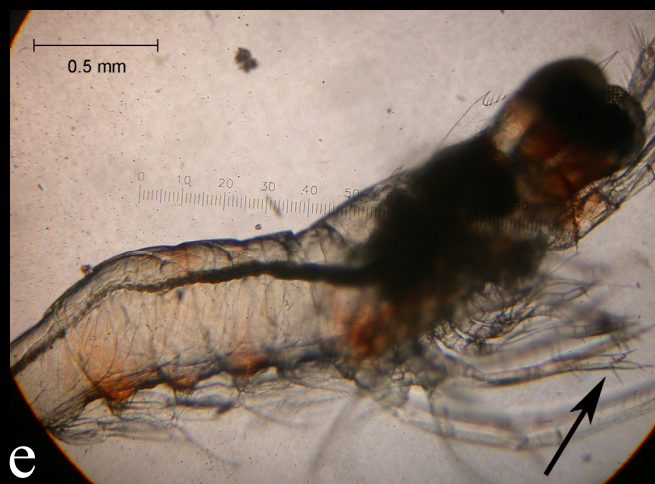

Supplement: Supplementary file 10 — Additional file 10: Figure S10: Zoea X. Lateral view (a). Four segments present in the antennular flagellum (arrows; b) and fifth rostral tooth present on the dorsal carina (c). Ten segments in the antennal flagellum (d) and rudimentary chelae present on the second pair of pereiopods (arrow; e). (PDF 972 KB) [file 40064_2014_1266_MOESM10_ESM.pdf]

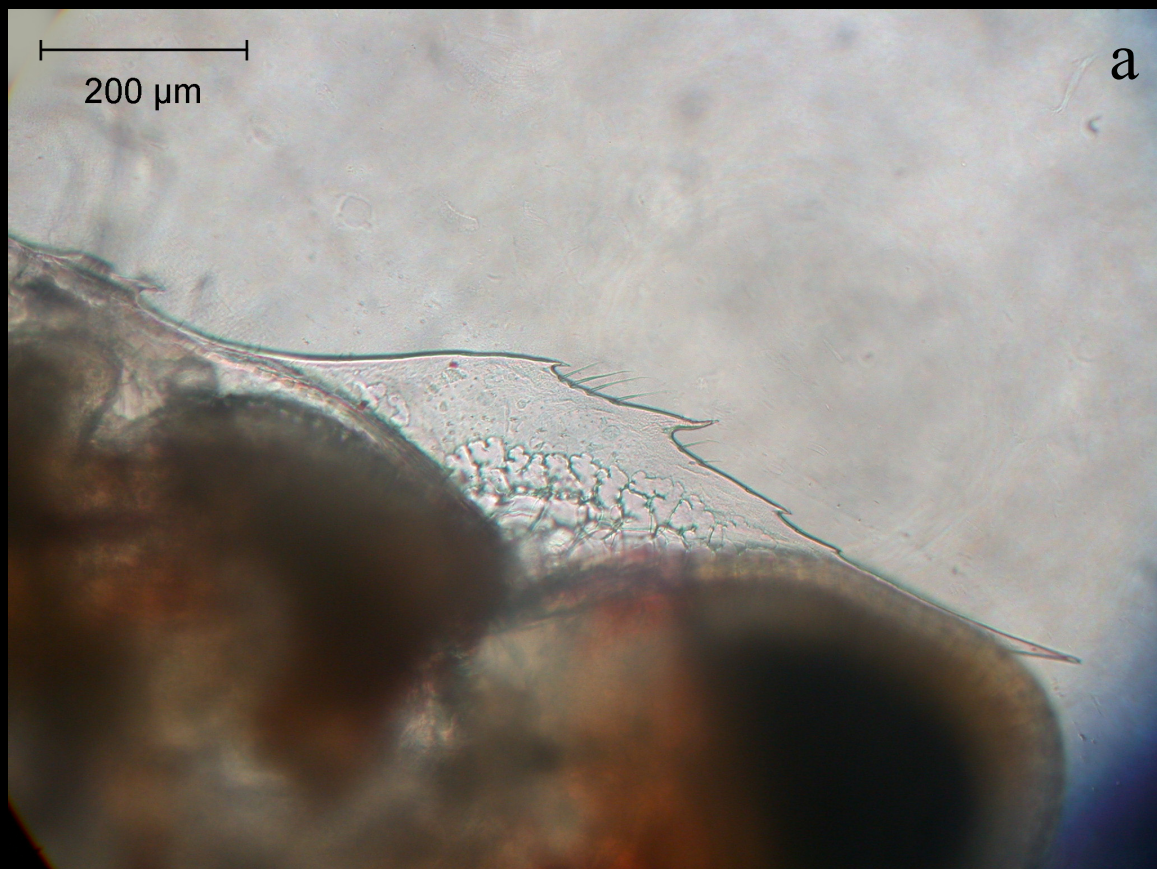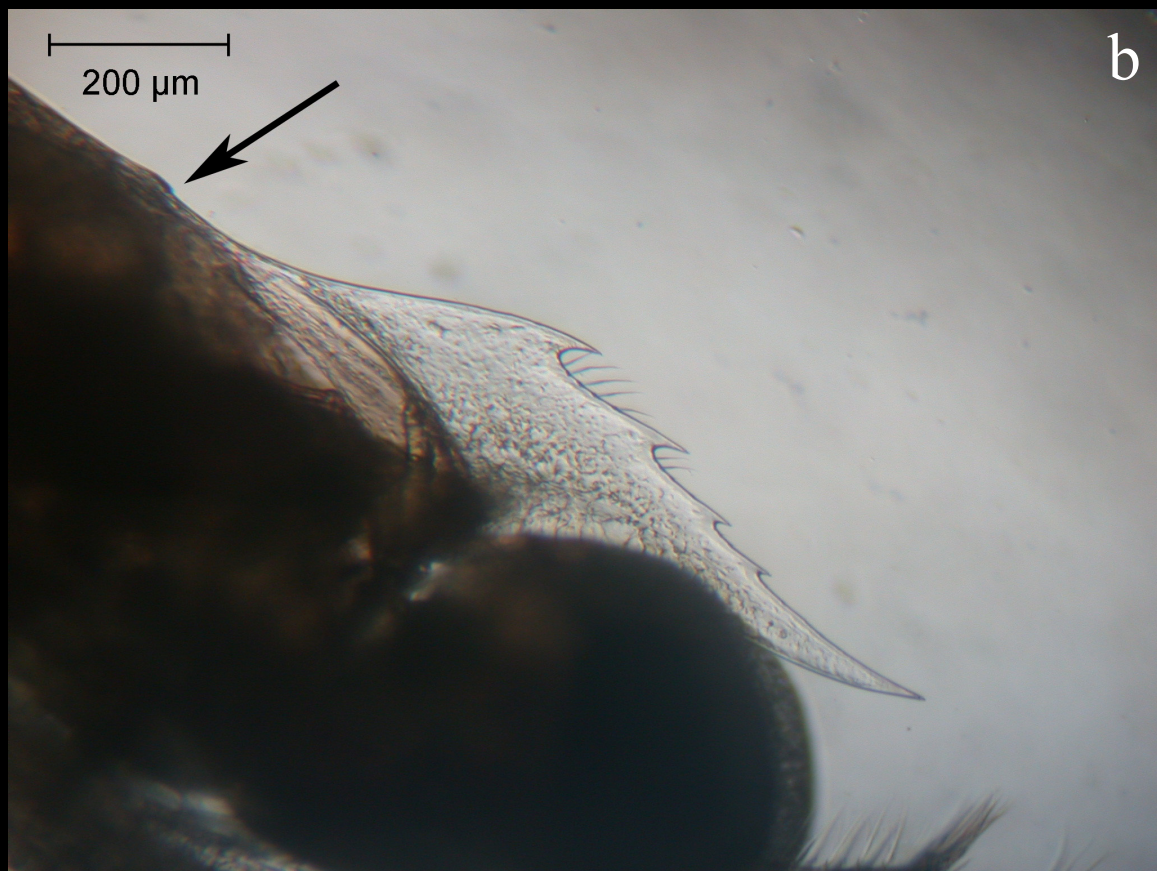

Supplement: Supplementary file 11 — Additional file 11: Figure S11: Zoea X showing variable rostral dentition. Individual with a post-orbital tooth (a) and an individual without a post-orbital tooth displaying a protrusion of the carapace (arrow; b). (PDF 1 MB) [file 40064_2014_1266_MOESM11_ESM.pdf]

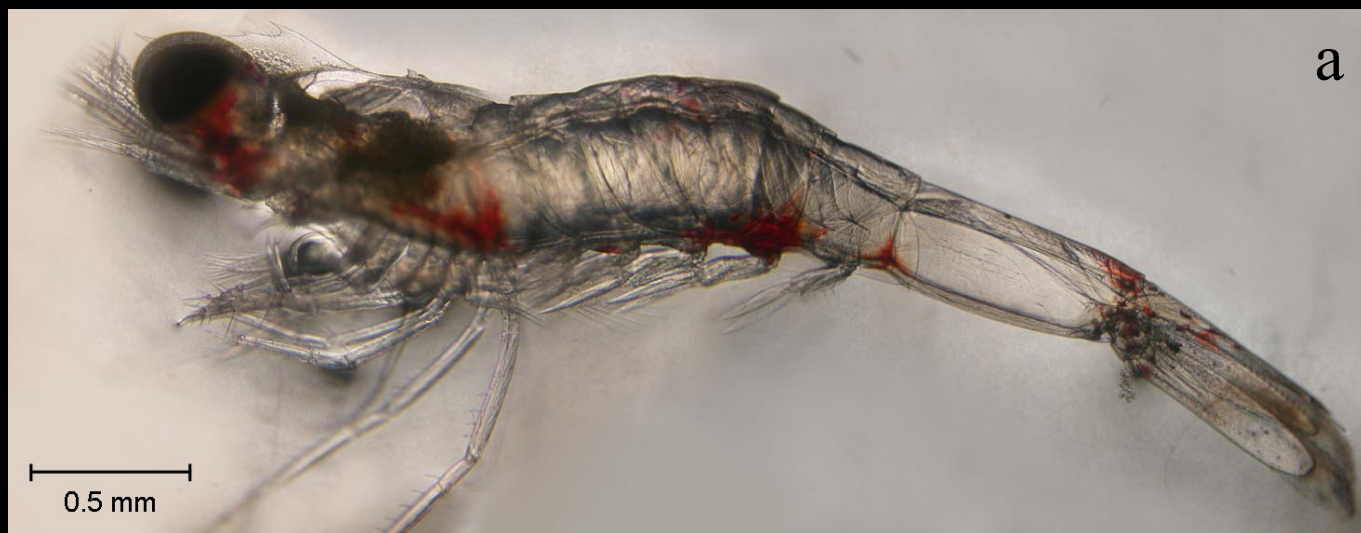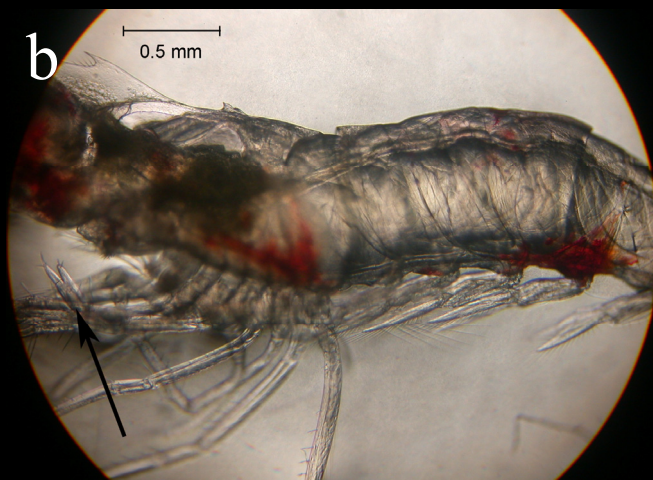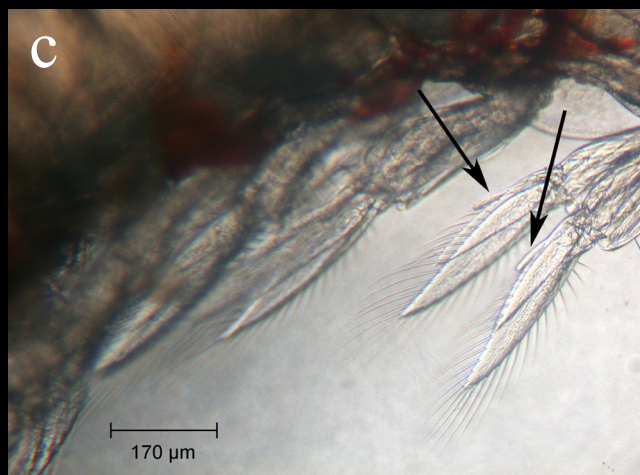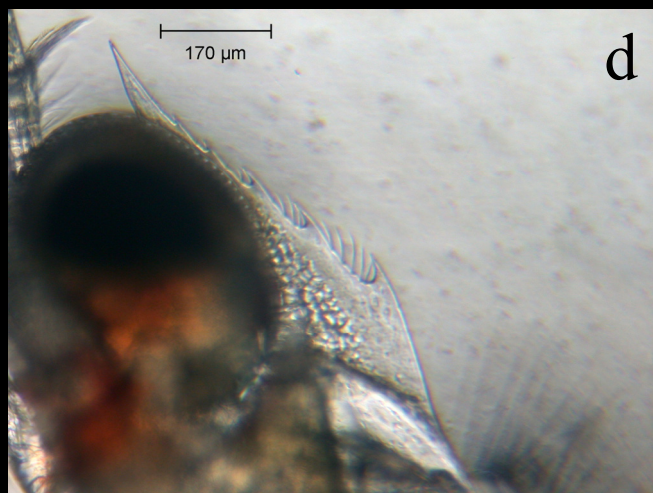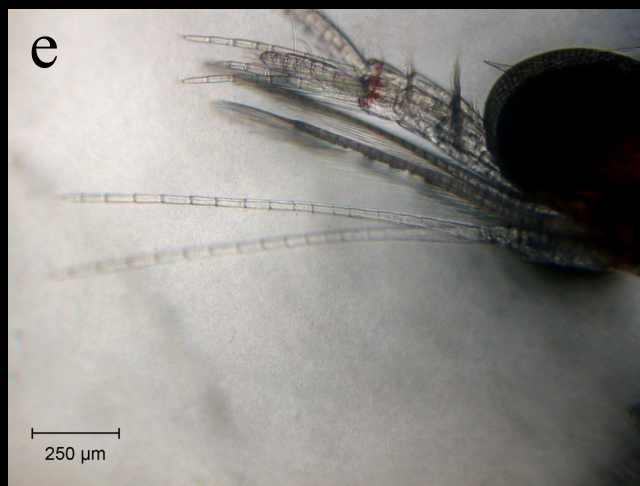

Supplement: Supplementary file 12 — Additional file 12: Figure S12: Zoea XI. Lateral view (a). Chelae present on the second pair of pereiopods are now larger (arrow; b) and appendix interna development is complete on all pleopods (arrows; c). This individual has 5 teeth on the dorsal carina (d). 14 – 18 segments present in the antennal flagellum (e). (PDF 1 MB) [file 40064_2014_1266_MOESM12_ESM.pdf]

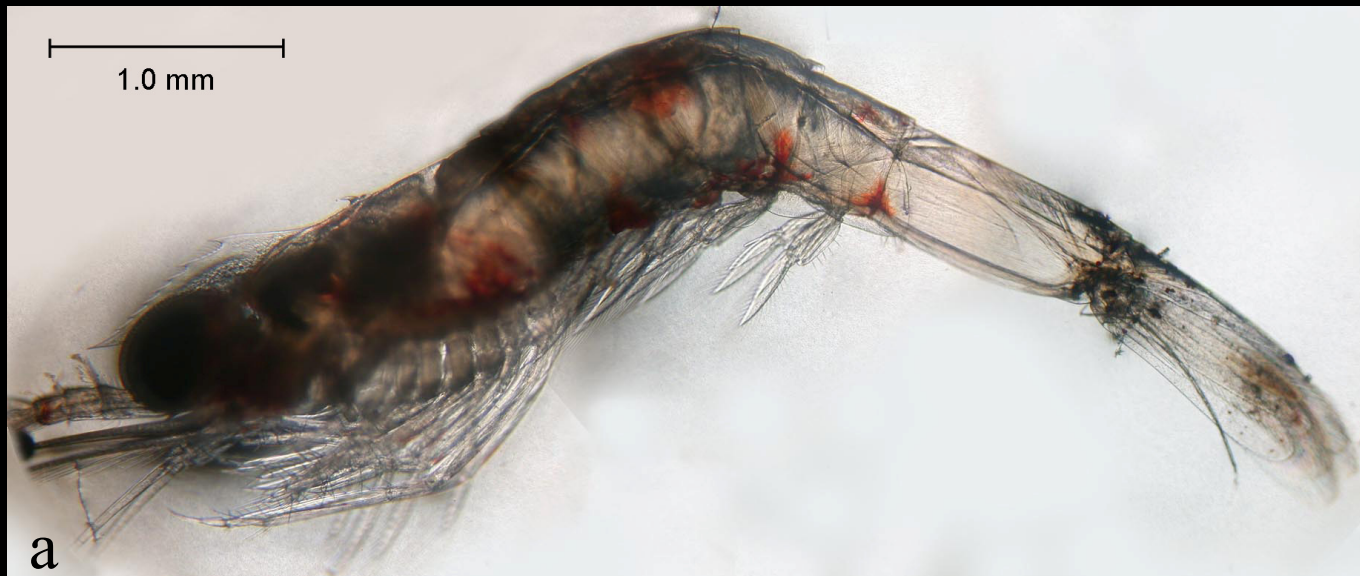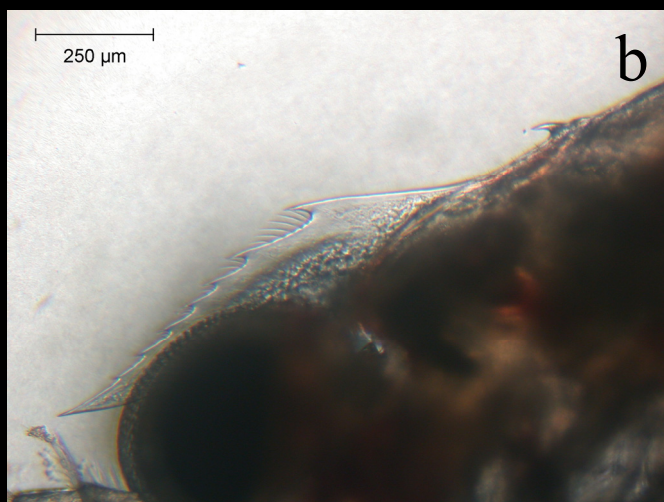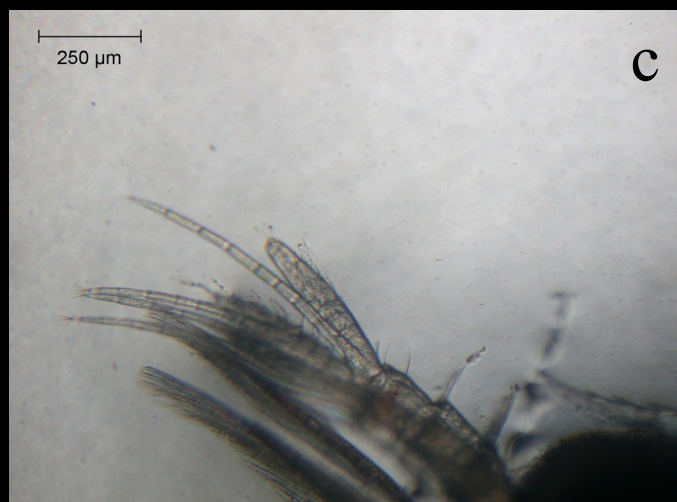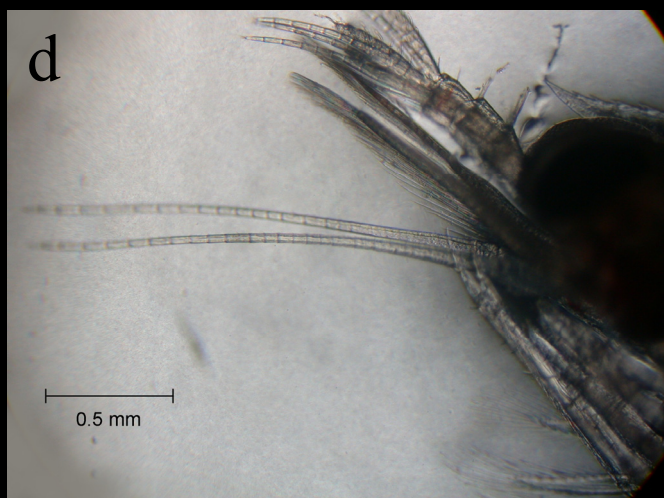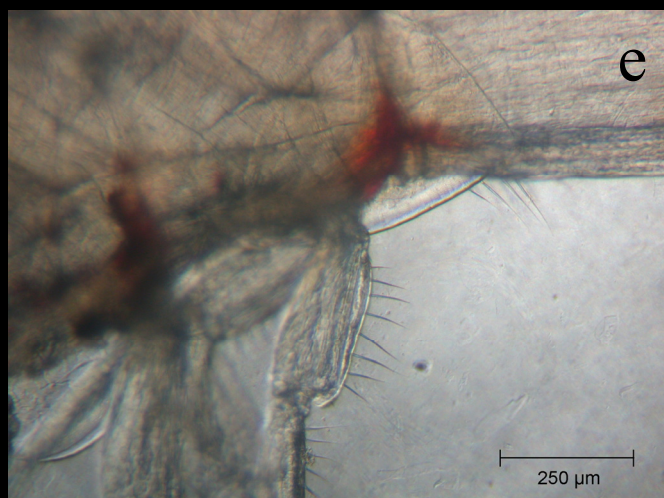

Supplement: Supplementary file 13 — Additional file 13: Figure S13: Zoea XII. Lateral view (a). This individual has 8 teeth on the dorsal carina (b). 9 segments present in the antennular flagellum (c) and ~20 segments in the antennal flagellum (d). 8 setae present on the rear margin of the basal segment of the fifth pair of pleopods (e). (PDF 1 MB) [file 40064_2014_1266_MOESM13_ESM.pdf]

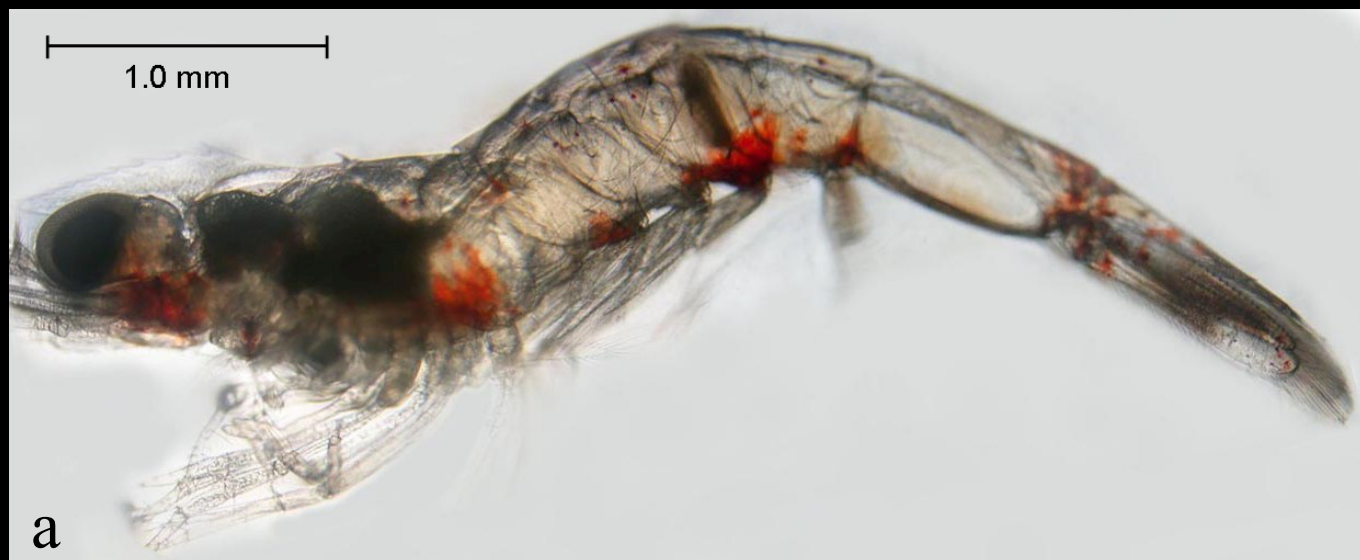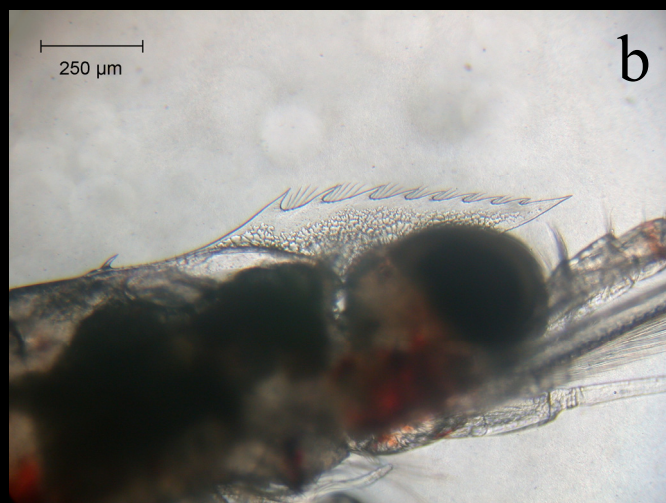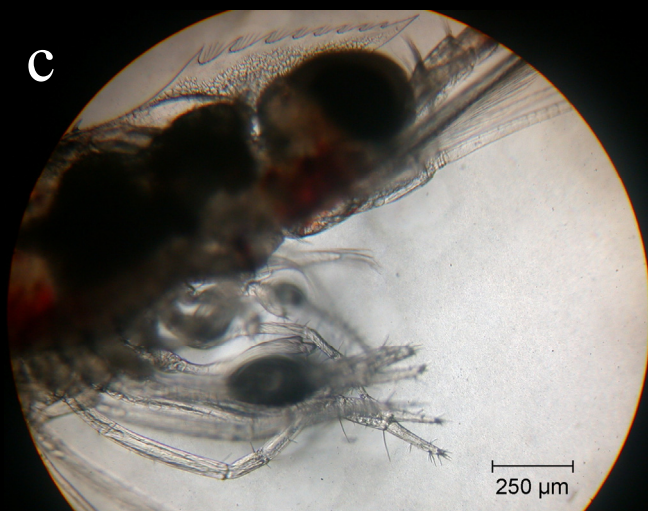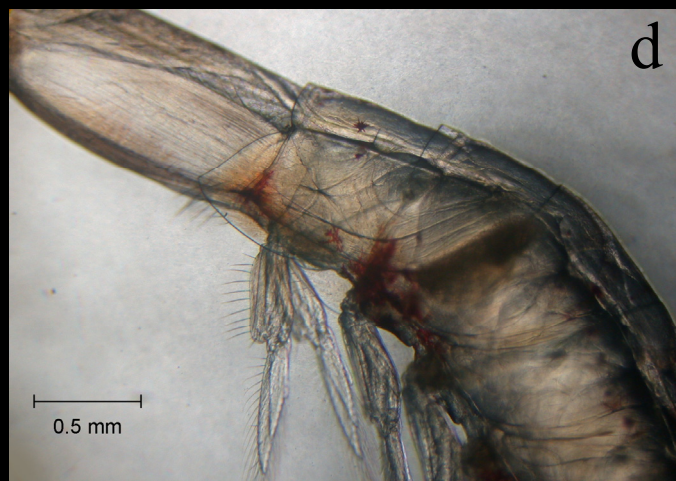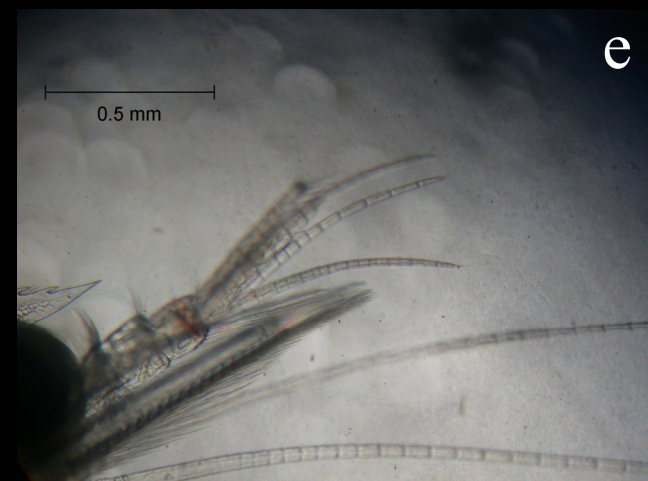

Supplement: Supplementary file 14 — Additional file 14: Figure S14: Zoea XIII. Lateral view (a). This individual has 9 teeth on the dorsal carina (b). Chelae present on the second pair of pereiopods are now further enlarged (c) and 11 setae present on the rear margin of the basal segment of the fifth pair of pleopods (d). 14 segments present in the antennular flagellum (e). (PDF 1 MB) [file 40064_2014_1266_MOESM14_ESM.pdf]

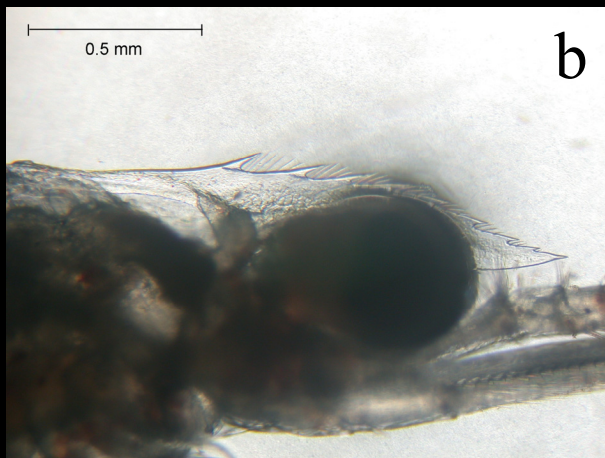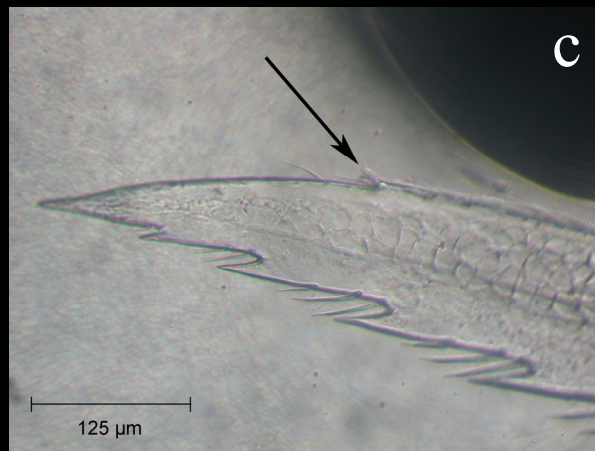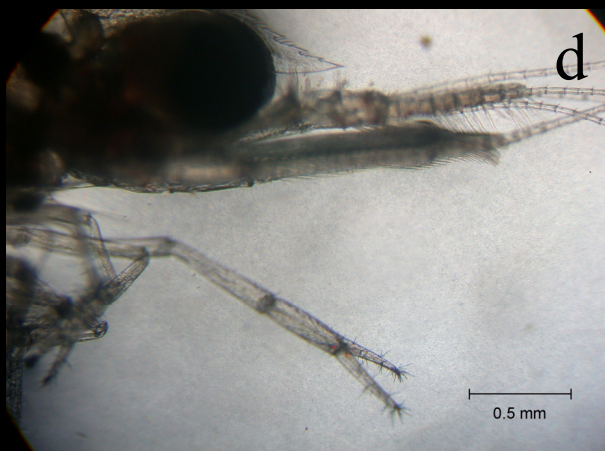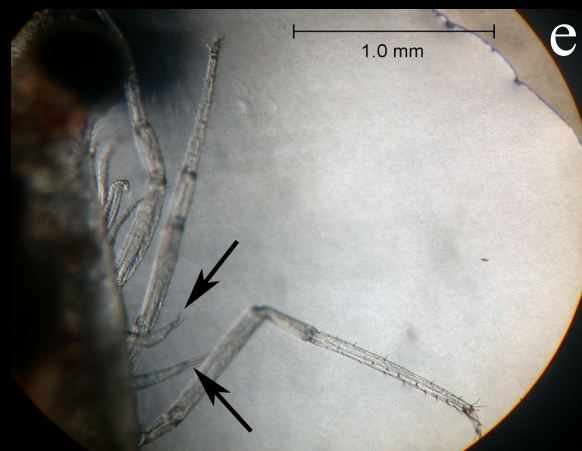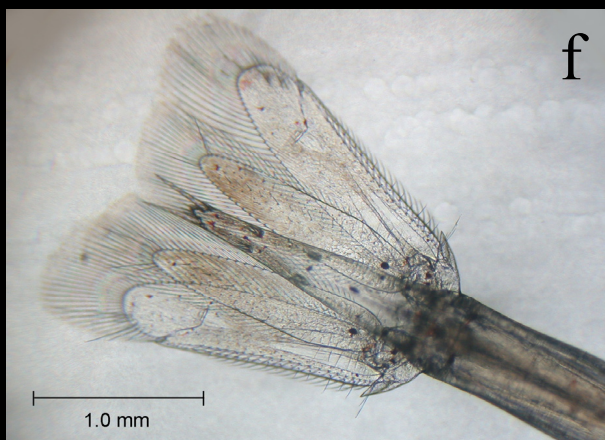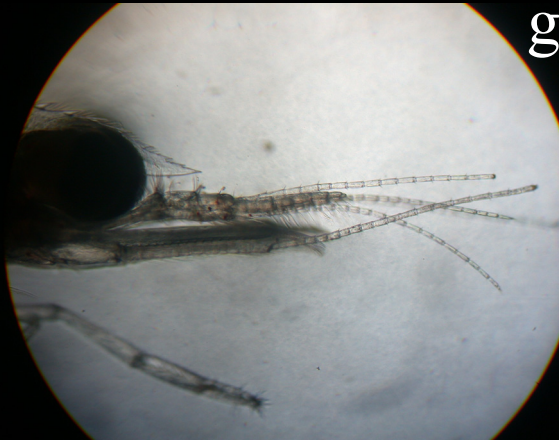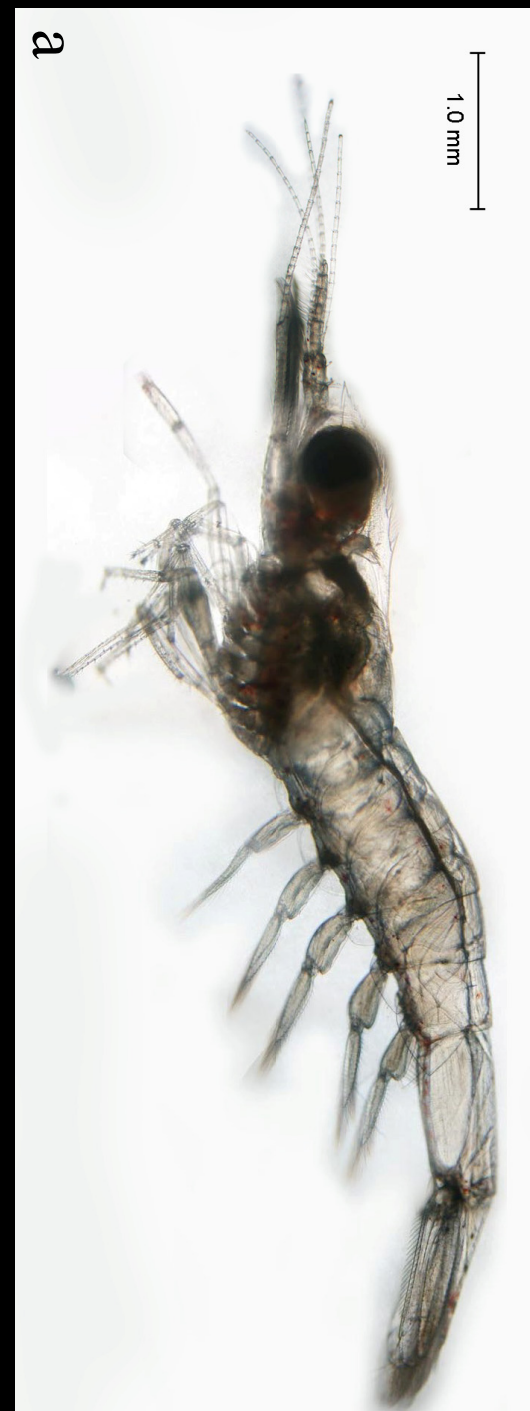

Supplement: Supplementary file 15 — Additional file 15: Figure S15: Decapodid. Lateral view (a). This individual has 8 teeth on the dorsal carina (b). The first rostral tooth on the ventral carina (arrow; c) and greatly enlarged second pair of pereiopods and chelae (d). Rudimentary natatory pereiopod exopodites (arrows; e) and triangular telson (f). 16+ segments present in the antennular flagellum (g). (PDF 1 MB) [file 40064_2014_1266_MOESM15_ESM.pdf]
